# Multimaterial 3D Printing of Soft and Stretchable Electronics

**DOI:** 10.1002/advs.202513208

**Published:** 2025-11-21

**Authors:** Omid Dadras‐Toussi, Bhoomija Hariprasad, Mohammad Reza Abidian

**Affiliations:** ^1^ Department of Biomedical Engineering University of Houston 3517 Cullen Blvd Houston TX 77204 USA

**Keywords:** additive manufacturing, carbon nanotubes, microelectronics, multimaterial 3D printing, two‐photon polymerization conducting polymers

## Abstract

The development of soft and stretchable microelectronics is critical for next‐generation flexible devices, biointerfaces, and microscale energy systems due to their unique electrical and mechanical properties. However, current 3D printing methods, particularly two‐photon polymerization (2PP), remain limited by low electrical conductivity, filler aggregation, and loss of optical transparency. Here, we present a multimaterial 2PP‐compatible resin that integrates the conducting polymer PEDOT:PSS and multi‐walled carbon nanotubes within a hydrogel PEGDA matrix to overcome these challenges. The optimized composite achieves a conductivity of 1.4 × 10⁵ S m^−1^ (≈10⁴‐fold improvement over pristine PEGDA), > 80% optical transmittance, and stable high‐resolution patterning. Directly printed microresistors and microcapacitors exhibit a specific capacitance of ≈667 F g^−1^, combining electric‐double‐layer and pseudocapacitive charge storage. The printed structures maintain ≈65% of their conductivity under 50% tensile strain and remain conductive after 3000 stretching cycles at 10% strain, with no delamination from PDMS. The composite also preserves geometry and adhesion across pH 3–10, confirming chemical robustness. This sequential multimaterial 2PP approach enables monolithic integration of conductive, insulating, and electroactive domains for flexible, stretchable, and chemically stable soft microelectronics, advancing scalable fabrication of biointerfaces, wearable devices, and microscale energy‐storage systems.

## Graphical abstract

A multimaterial resin for two‐photon polymerization integrates PEDOT:PSS and carbon nanotubes to enable direct 3D printing of conductive, insulating, and electroactive microstructures. The hybrid exhibits high conductivity, optical transparency, and stability under strain and pH variation, advancing scalable fabrication of flexible soft electronics and bioelectronic microsystems.

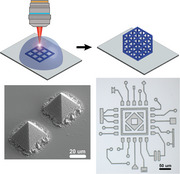

## 1 Introduction

Organic electronics have garnered significant attention in diverse areas of micro‐ and nanotechnology, including electronics,^[^ ^1^ ^]^ energy storage,^[^ ^2^ ^]^ and biomedical science,^[^ ^3^, ^4^ ^]^ owing to their unique combination of high electrical and soft mechanical properties,^[^ ^5^ ^]^ chemical stability,^[^ ^6^ ^]^ and biocompatibility.^[^ ^7^ ^]^ Various techniques have been explored for fabricating 3D organic electronic structures, including ink jet printing,^[^ ^8^ ^]^ soft lithography patterning,^[^ ^9^ ^]^ extrusion printing,^[^ ^10^ ^]^ electrodynamic printing,^[^ ^11^ ^]^ digital light processing,^[^ ^12^ ^]^ and electrochemical patterning.^[^ ^13^ ^]^ However, these techniques often suffer from limited spatial resolution, multistep procedures, and high processing costs.^[^ ^14^ ^]^

In contrast, direct laser writing (DLW) via two‐photon polymerization (2PP) is a maskless state‐of‐the‐art light‐based additive manufacturing technique, capable of fabricating complex micro‐ and nanoscales architectures with spatial resolution down to ≈15 nm.^[^ ^15^, ^16^ ^]^ During 2PP, a femtosecond‐pulsed near‐infrared laser induces simultaneous two‐photon absorption within a transparent photosensitive resin, initiating highly localized polymerization or crosslinking (i.e., negative photoresist). Fine features size below 100 nm are achievable through rigorous optimization of resin formulations, writing speed, and laser parameters.^[^ ^17^ ^]^ Incorporation of stimuli‐responsive nanomaterials and biomolecules within 2PP‐compatible resin has enabled fabrication of functional microstructures^[^ ^18^ ^]^ such as remote‐controlled micromachines,^[^ ^19^ ^]^ photoluminescent devices,^[^ ^20^ ^]^ and biosensors.^[^ ^21^, ^22^, ^23^, ^24^ ^]^

Recent research has focused on enhancing the conductivity of 2PP‐fabricated microstructures by integrating conductive fillers. Inorganic conductive fillers such as metallic nanoparticles^[^ ^18^ ^]^ and carbon nanotubes,^[^ ^25^ ^]^ as well as organic conductive materials including organic semiconductors^[^ ^26^, ^27^ ^]^ have been explored. While a high concentration of conductive metallic nanoparticles (>10 wt.%) can significantly increase electrical conductivity, their aggregation often leads to fabrication of low‐quality planar microstructures with structural inhomogeneities and reduced mechanical integrity.^[^ ^18^ ^]^ For instance, Shukla et al. incorporated metallic salt HAuCl_4_ into SU‐8 resins, achieving conductivity values ≈2.5 × 10⁴ S m^−1^; however, particle aggregation resulted in compromised structural quality.^[^ ^28^ ^]^

Carbon‐based nanofillers, such as multi‐walled carbon nanotubes (MWCNTs)^[^ ^25^ ^]^ and graphene oxide,^[^ ^29^ ^]^ offer only lower concentrations (<0.2 wt.%) due to large particle agglomerates, and produce structures with higher aspect ratios and uniformity. Nevertheless, their inherent tendency to agglomerate and relatively moderate electrical conductivities (typically <10^2^ S m^−1^) pose considerable limitations. For example, Xiong et al. incorporated a maximum of 0.2 wt.% MWCNTs into thiol‐acrylate resins to fabricate 3D conductive structures via 2PP, achieving conductivities of ≈46.8 S m^−1^. However, limited doping levels and moderate conductivity have constrained practical applicability.^[^ ^25^ ^]^

Organic electronics (OEs), specifically poly(3,4‐ethylenedioxythiophene) (PEDOT), represent an attractive alternative due to their unique mixed electronic‐ionic conduction property, excellent mechanical flexibility, and high biocompatibility.^[^ ^5^, ^30^, ^31^, ^32^, ^33^ ^]^ Building upon these findings and addressing remaining limitations, we rationalize the combined use of OEs and MWCNTs in this study. We hypothesized that a synergistic combination of these two fillers would overcome miscibility constrains, thereby significantly enhancing electrical conductivity, delivering enhanced electron transport pathways, and maintaining excellent optical transparency. Here, we have formulated a novel transparent, photosensitive resin suitable for fabricating organic electronic and multi‐walled carbon nanotube microstructures (OEMWCNTMSs) with relatively smooth surfaces via 2PP laser direct writing. For the first time, we demonstrate that the direct and simultaneous incorporation of two conductive fillers 0.4 wt.% OE and 0.15 wt.% MWCNTs into a poly(ethylene glycol) diacrylate ‐based resin yields a dramatic enhancement in the electrical conductivity of 2PP‐fabricated microstructures, exceeding ten orders of magnitude relative to the pristine matrix. The resulting OEMWCNTMSs achieve a specific conductivity of ≈2.5 × 10⁵ S m^−1^ wt.%^−1^, surpassing all previously reported conductive resin systems, while maintaining high structural resolution, uniformity, and optical transparency.

We successfully demonstrated comprehensive structural, electrical, electrochemical, and stability characterization of various OEMWCNT microstructures, including micro‐scale printed circuit boards and microelectrodes. The resulting microcapacitors exhibited exceptional energy‐storage performance, achieving a specific capacitance of ≈667 F g^−1^, exceeding previously reported values for 2PP‐fabricated microcapacitors derived from organic electronic and carbon nanotube‐based resins. Furthermore, we developed a multimaterial 3D microfabrication strategy leveraging sequential 2PP to enable spatially controlled patterning of conductive, insulating, and electroactive domains within an integrated monolithic architecture. The optimized composite microstructures exhibited high electrical conductivity (≈1.4 × 10⁵ S m^−1^) and excellent optical transparency (≈82% transmittance), along with mechanical durability and stretchability, retaining conductivity under cyclic 10% strain for up to 3000 cycles, and chemical robustness against pH fluctuations from 3 to 10.

Together, these properties establish a new class of soft, conductive, and stretchable microstructures fabricated via two‐photon polymerization, offering a versatile platform for flexible microelectronics, microscale energy storage, wearable biosensors, and neural interface technologies, and marking a significant advancement toward fully integrated organic bioelectronic microsystems.

## 2 Results and Discussion

**Figure 1:**
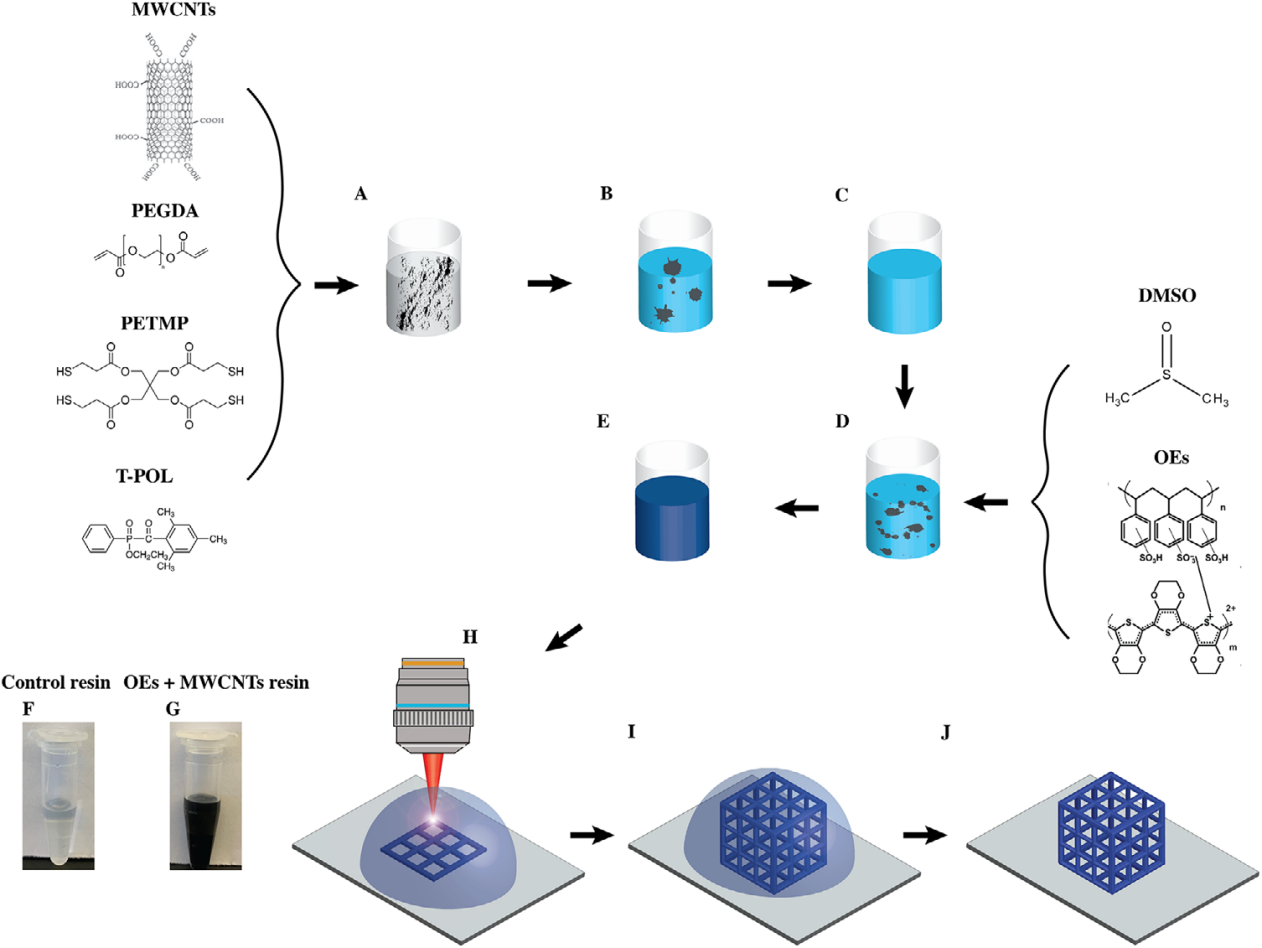
Schematic representation of resin formulation and two‐photon polymerization (2PP) fabrication of conductive polymer‐multiwalled carbon nanotube microstructures (OEMWCNTMSs). (A) Chemical structures of resin components: acid‐functionalized multiwalled carbon nanotubes (MWCNTs), poly(ethylene glycol) diacrylate (PEGDA), pentaerythritol tetrakis(3‐mercaptopropionate) (PETMP), and ethyl (2,4,6‐trimethylbenzoyl) phenylphosphinate (T‐POL). (B,C) First step resin preparation: MWCNTs, PEGDA, PETMP, and T‐POL are magnetically stirred to ensure uniform MWCNT dispersion and subsequently centrifuged to remove large aggregates. (D) Second step resin preparation: addition of dimethyl sulfoxide (DMSO) and organic electronic OE into the resin mixture. (E) Resin after additional stirring, achieving homogeneity with OE uniformly dispersed. (F,G) Images comparing the control resin (F, without fillers) and conductive composite resin (G, containing MWCNTs and OEs), demonstrating homogeneity. (H–J) Illustration of the 2PP lithography process: femtosecond laser‐induced crosslinking at the focused region of the resin droplet results in the fabrication of microstructures layer‐by‐layer, based on computer‐designed 3D patterns.

**Figure** **1** illustrates the chemical components of the composite resin and the detailed 2PP fabrication procedure for OEMWCNTMSs. The formulation and preparation of a homogeneous, 2PP‐compatible photosensitive resin are crucial steps for fabricating OEMWCNTMSs (see Experimental Section, Tables S1–S4, Supporting Information). Poly(ethylene glycol) diacrylate (PEGDA) and ethyl (2,4,6‐trimethylbenzoyl) phenylphosphinate (TPO‐L) were selected as the polymer crosslinker and photoinitiator, respectively. PEGDA is widely employed in 2PP resins due to its suitability for high‐resolution printing, favorable mechanical properties, and biocompatibility.^[^ ^34^, ^35^, ^36^ ^]^ TPO‐L was chosen based on its water solubility, biocompatibility,^[^ ^37^, ^38^ ^]^ and optimal absorption range (600–810 nm) aligning with the laser wavelength used (780 nm).^[^ ^39^, ^40^ ^]^

The resin preparation comprised two main steps to ensure uniform dispersion of carbon nanotubes (CNTs) and the organic electronic (OE). Initially, MWCNTs, pentaerythritol tetrakis(3‐mercaptopropionate) (PETMP), TPO‐L, and PEGDA were mixed (Figure 1A) and magnetically stirred for 12 h to achieve homogeneous dispersion of MWCNTs (Figure 1B). PETMP was specifically chosen to disperse the MWCNTs uniformly and prevent aggregation by interacting via branched thiol groups with the functionalized carboxyl groups on MWCNTs. Acid‐purified short‐length MWCNTs (10–30 µm, with 3.86 wt.% carboxyl groups) were employed to maximize uniform dispersion without significant aggregation. Following the stirring step, large MWCNT aggregates were removed through centrifugation (Figure 1C; Figure S1, Supporting Information), achieving a maximum uniform dispersion concentration of 0.15 wt.% (Figure S2, see Resin Stability and Homogeneity in Supporting Information). There is a recognized trade‐off between MWCNT length and electrical properties within polymeric matrices.^[^ ^41^ ^]^ Shorter MWCNTs, enable higher filler concentrations to be uniformly dispersed within the resin, while exhibiting lower electrical conductivities. This increased concentration compensates for any reduced per‐particle conductivity.^[^ ^25^ ^]^ Furthermore, shorter MWCNTs promote the formation of a more homogeneous resin, a crucial factor for achieving high resolution and structural fidelity in the 2PP fabrication process. Conversely, longer MWCNTs tend to aggregate more readily,^[^ ^42^ ^]^ negatively affecting resin uniformity and causing laser‐induced structural deformations and compromised microstructural resolution during printing.

**Figure 2:**
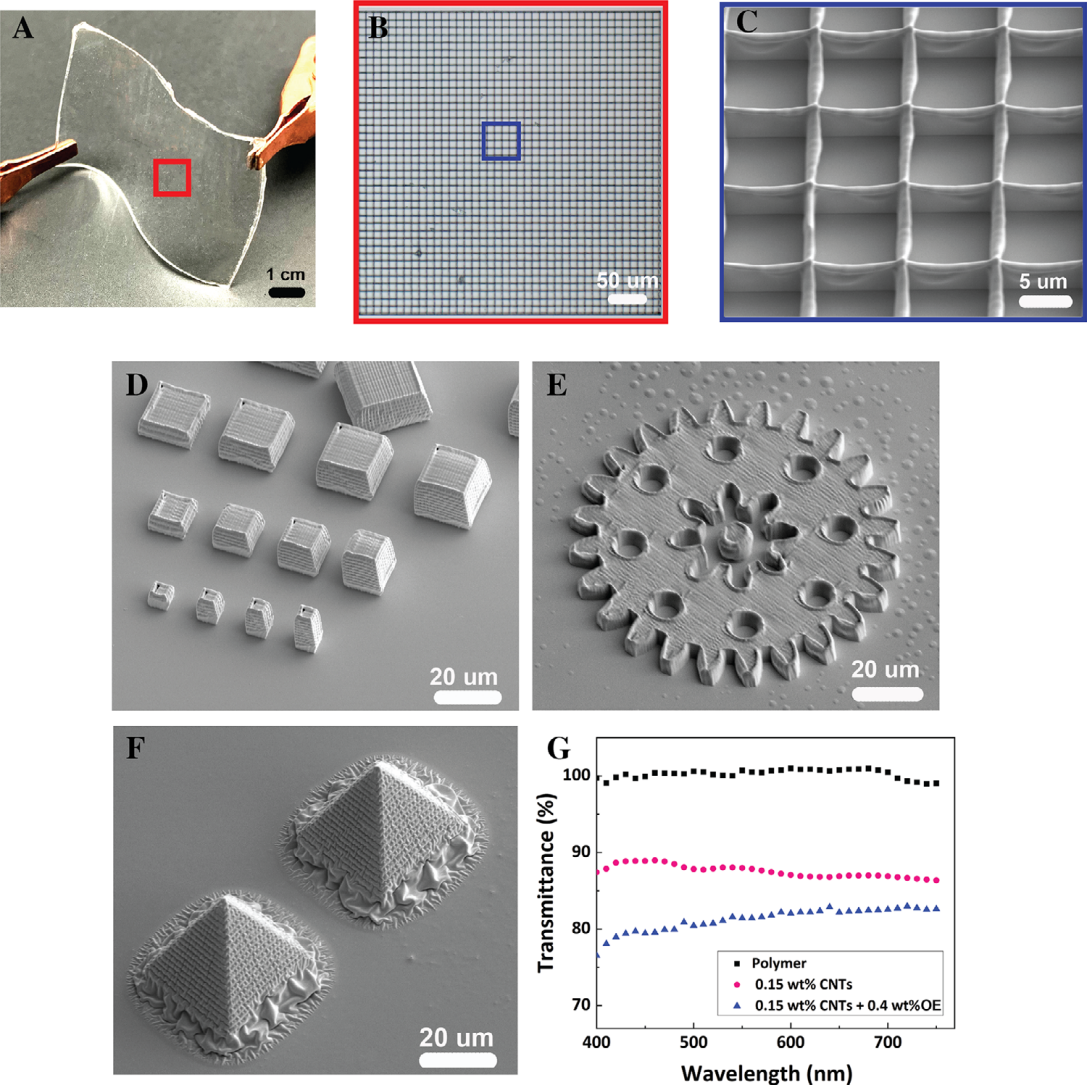
Two‐photon polymerization (2PP) fabrication and optical characterization of conductive polymer‐MWCNT composite microstructures (OEMWCNTMSs). (A) Photograph illustrating flexible poly(dimethylsiloxane) (PDMS) substrate demonstrating substrate flexibility. (B,C) Optical microscopy (B) and SEM (C) images showing uniformity and precise geometry of a 40 × 40 microgrid. (D) SEM image of a systematically fabricated micro‐cubic array highlighting scalability and precision. (E) SEM image of a detailed micro‐gear demonstrating complex structural fidelity (see also Figures S2A–C, Supporting Information). (F) SEM image of micro‐pyramids exhibiting excellent structural integrity. (G) Transmittance spectra comparing pristine polymer resin (PEGDA), resin containing 0.15 wt.% MWCNTs, and resin containing both 0.15 wt.% MWCNTs and 0.4 wt.% OE, illustrating high optical transparency suitable for optoelectronic applications.

In the second step, dimethyl sulfoxide (DMSO) and poly(3,4‐ethylenedioxythiophene)‐poly(styrenesulfonate) (PEDOT:PSS) were added to the resin mixture (Figure 1D) and stirred for an additional 2 h (Figure 1E). DMSO enhanced the miscibility of PEDOT:PSS (i.e., OE) within the resin^[^ ^43^ ^]^ and further improved conductivity.^[^ ^44^, ^45^, ^46^, ^47^ ^]^ The maximum homogeneous concentration of OE incorporated into the resin was 0.4 wt.% (Figure S3A–D, Supporting Information). Ultimately, the final resin comprised both MWCNTs and OE uniformly dispersed within the PEGDA‐based composite (Figure 1F–G) and was stable for ≈6 hrs at room temperature (Figure S4A–F, Supporting Information). This carefully optimized homogeneous resin formulation ensured compatibility with the highly sensitive 2PP lithography process. The inclusion of PETMP and DMSO as dispersants for MWCNTs and OE, respectively, was critical to achieving the high degree of resin homogeneity required. Additionally, removing large MWCNT aggregates through centrifugation prevented laser‐induced structural defects during 2PP fabrication. During the 2PP process, a femtosecond laser beam (center wavelength of 780 nm, pulse width of 130 nm, repetition rate of 80 MHz, and power of 1.7 mW) selectively crosslinked the resin in a layer‐by‐layer manner according to predefined 3D computer‐generated patterns, resulting in precisely structured OEMWCNTMSs fabricated on glass substrates (Figure 1H–J) and flexible poly(dimethylsiloxane) (PDMS) substrates (**Figure** **2**A). Beyond glass and PDMS, the 2PP workflow used here is compatible with a wide set of substrates provided that optical access at the writing wavelength and adequate interfacial adhesion are ensured. In practice, we have employed standard pretreatments (e.g., oxygen plasma and acrylate silanization/tethering) that enable robust adhesion on Si/SiO_2_, polyimide (Kapton), parylene‐C, PET/PC, and metal‐coated surfaces (with thin Ti/Au adhesion layers). Because polymerization occurs within the focal volume of the resin, the method also extends to nonplanar and freeform surfaces using immersion objectives and local refocusing/dip‐in writing, consistent with prior demonstrations of high‐resolution 2PP on freeform curved substrates.^[^ ^48^ ^]^

Figure 2B–F illustrates various 3D printed OEMWCNTMSs via 2PP from the optimized composite resin containing 0.15 wt.% MWCNTs and 0.4 wt.% OE. Specifically, these structures include a 40 × 40 microgrid (Figure 2B,C), a systematically fabricated micro‐cubic array (Figure 2D), a detailed micro‐gear (Figure 2E; Figure S5 Supporting Information), and a micro‐pyramid array (Figure 2F). These optical and SEM micrographs collectively highlight the uniformity, precise geometry, scalability, and exceptional structural integrity of the fabricated OEMWCNTMSs. Additionally, optical transparency of the composite resin was characterized with respect to the concentrations of OE and MWCNTs (Figure 2G). The transmittance spectra reveal that the pristine polymer (PEGDA, black squares) maintains excellent optical clarity across the visible spectrum (400–750 nm), with transmittance values near 100%. Incorporating 0.15 wt.% MWCNTs (pink circles) introduces modest reductions in transparency, attributable to intrinsic absorption and scattering effects, yet maintaining high transparency (>85%). Notably, the final optimized formulation containing both 0.15 wt.% MWCNTs and 0.4 wt.% OE (blue triangles) continues to exhibit exceptional optical transmittance exceeding 80% throughout the visible range. This robust optical transparency (>80% across 400–800 nm), despite incorporating conductive nanomaterials, exceeds the minimum requirements for optical biointerfaces (≥60%) and optoelectronic systems (≥70–75%),^[^ ^49^, ^50^ ^]^ highlighting the resin's suitability for advanced photonic devices^[^ ^50^ ^]^ and soft neural interfaces,^[^ ^49^ ^]^ while remaining fully compatible with precise 2PP fabrication techniques.

**Figure 3:**
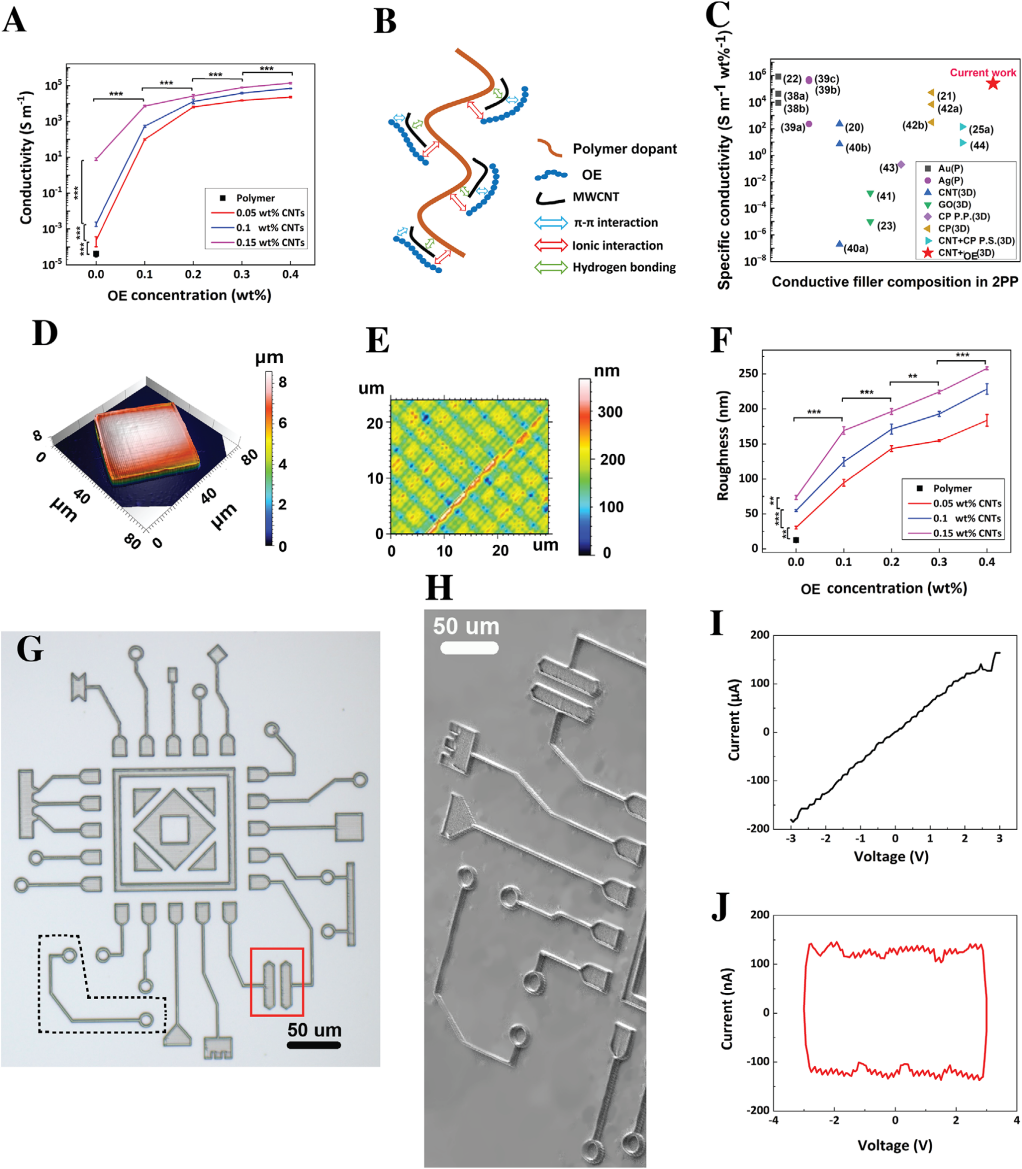
Electrical, morphological, and functional performance of OEMWCNTMSs fabricated using 2PP. (A) Electrical conductivity of microstructures as a function of OE concentration (0–0.4 wt.%) at three MWCNT loadings (0.05, 0.1, and 0.15 wt.%). Conductivity increased with both MWCNT and OE content, with significant enhancements observed at higher concentrations (*n* = 5, *p* < 0.001). (B) Schematic illustration of proposed synergistic interactions between OE, polymer dopant, and functionalized MWCNTs in PEGDA matrix, including *π*–*π* stacking, ionic interactions, and hydrogen bonding. (C) Comparison of specific conductivity (conductivity per wt.%) for 2PP‐fabricated microstructures incorporating different conductive fillers. Gold (Au)^[^ ^28^, ^59^, ^60^ ^]^ black squares, silver (Ag)^[^ ^61^, ^62^, ^63^ ^]^ purple circles, carbon nanotubes (CNT)^[^ ^25^, ^64^, ^65^ ^]^ blue up triangles, graphene oxide (GO)^[^ ^29^, ^66^ ^]^ green down triangles, conducting polymer (CP)^[^ ^26^, ^67^, ^68^ ^]^ orange left triangle, conducting polymer post polymerization step (CP P.P.)^[^ ^69^ ^]^ purple diamond, carbon nanotube and conducting polymer post soaking step (CNT+CP P.S.)^[^ ^34^, ^70^ ^]^ green right triangle, this study: carbon nanotube and organic electronic (CNT+OE) red star. (P) represents planar structure and (3D) represents 3D structure. The hybrid OE + MWCNT (CNT+OE) composite (red star) achieved the highest specific conductivity among all reported 3D structures. (D and E) Materials confocal Microscopy color‐coded height map showing surface texture and surface roughness of a microstructure fabricated with 0.15 wt.% MWCNTs and 0.4 wt.% OE, respectively, exhibiting uniform surface texture. (F) Surface roughness (Rrms) as a function of OE concentration at various MWCNT contents. Increased OE content led to statistically significant surface roughening across all MWCNT levels (*n* = 5, *p* < 0.01, *p* < 0.001). (G) Optical image of a fully printed µPCB incorporating various passive circuit elements. The dashed box highlights the printed resistor, and the red box indicates the printed microcapacitor. (H) SEM image of the 3D microprinted circuit board (µPCB) on PDMS, illustrating the spatial resolution and vertical profile of conductive features shown in (G). (I) I–V characteristics of the printed microresistor 150 × 2 µm × 1 µm (dashed box) showing linear ohmic behavior with a conductance of 106.52 ± 9.31 µS for the. (J) Hysteresis loop of a printed microcapacitor (red box) exhibiting a rectangular I–V profile, characteristic of ideal capacitive behavior, with a specific capacitance of 667 ± 0.1 F g^−1^. Data shown as mean ± S.E.M, (*n* = 5). Statistical significance denoted as *p* < 0.01, *p* < 0.001.

To investigate the electrical properties of OEMWCNTMSs, first we designed and fabricated microbars with length 125 µm, width 20 µm, and height 5 µm, which connected two parts of a gold‐coated glass (Figure S6A, Supporting Information). Semiconductor Device Parameter Analyzer (B1500A, Keysight) was used to measure current–voltage (I–V graph) and to calculate the electrical conductivity (Figure S6B–D, Supporting Information) (See Electrical Measurement in Experimental Section). **Figure** **3**A shows a comprehensive graph of electrical conductivity of OEMWCNTMSs as a function of different MWCNT (CNTs: 0, 0.05, 0.1, and 0.15 wt.%) and OE (OE: 0, 0.1, 0.2, 0.3, and 0.4 wt.%) concentrations in the resin. As shown by the black square, the polymer PEGDA microstructures (without CNTs and OE) were not conductive, while addition of 0.05 wt.% MWCNTs (without OE) into the resin significantly increased the electrical conductivity of polymer microstructures from 4.1× 10^−5^ ± 1.3 × 10^−6^ S m^−1^ to 2.3 × 10^−4^ ± 1.3 × 10^−4^ S m^−1^ (*n* = 5, *p* < 0.001). Moreover, the electrical conductivity significantly increased to 8.2 ± 1.4 S m^−1^ (*n* = 5, *p* < 0.001) by increasing the concentration of MWCNTs to 0.15 wt.%. There was statistically a significant difference in electrical conductivity of MWCNT microstructures fabricated with 0.05, 0.1, and 0.15 wt.% (2.3 × 10^−4^ ± 1.3 × 10^−4^ S m^−1^, 1.9 × 10^−3^ ± 5.2 × 10^−4^ S m^−1^, and 8.2 ± 1.4 S m^−1^, respectively, (*n* = 5, *p* < 0.001)). Furthermore, by incorporation of 0.1, 0.2, 0.3, and 0.4 wt.% of OE into the resin at 0.15 wt.% MWCNTs, the electrical conductivity of OEMWCNTMSs dramatically increased to 7.4 × 10^3^ ± 6.9 × 10^2^ S m^−1^, 2.7 × 10^4^ ± 6.5 × 10^3^ S m^−1^, 7.9 10^4^ ± 4.4 × 10^3^, and 1.4 × 10^5^ ± 1.3 10^4^ S m^−1^, respectively. There was statistically significant different in electrical conductivity of OEMWCNTMSs fabricated with 0.1, 0.2, 0.3, and 0.4 wt.% of OE (*n* = 5, *p* < 0.001). It is noteworthy that presence of 0.15 wt.% MWCNTs and 0.4 wt.% OE in the resin resulted in a drastic improvement of electrical conductivity over 10 orders of magnitude, (from 4.1× 10^−5^ ± 1.3 × 10^−6^ S m^−1^ to 1.4 × 10^5^ ± 1.3 10^4^ S m^−1^ (*n* = 5, *p* < 0.001)). Furthermore, there was statistically significant difference between electrical conductivity of microstructures fabricated from resins with 0.15 wt.% MWCNTs (8.2 ± 1.4 S m^−1^), 0.4 wt.% OE (1.6 × 10^4^ ± 4.5 × 10^3^ S m^−^1), and 0.15 wt.% MWCNTs and 0.4 wt.% OE (1.4 × 10^5^ ± 1.3 10^4^ S m^−1^) (Figure S8, Supporting Information). Significantly higher conductivity of OE microstructures compared to MWCNT microstructures can be ascribed to presence of not only OE in the cross‐linked polymer chains that provides both ionic and electronic pathways along the polymer chains,^[^ ^31^ ^]^ but also conductivity enhancing agent DMSO^[^ ^44^, ^51^ ^]^ (also acts as the miscible agent) presumably due to reducing columbic interactions between OE and polymer dopant,^[^ ^52^ ^]^ changing conformation and reorienting of OE chains,^[^ ^43^ ^]^ and removing insulating polymer dopant.^[^ ^53^ ^]^ It is noteworthy that synergistic impact of OE and MWCNTs in electrical conductivity of OEMWCNTMSs can be explained by *π*–*π* interactions between OE and MWCNTs, causing more delocalized charges formed on OE chains, which facilitate charge density transfer from OE to MWCNTs and enhance the electrical conductivity (Figure 3B).^[^ ^54^, ^55^, ^56^, ^57^, ^58^ ^]^

To evaluate the efficiency of different conductive fillers in 2PP‐fabricated microstructures, we analyzed specific conductivity, defined as electrical conductivity normalized by filler weight percentage (S m^−1^ wt.%^−1^) across representative materials (Figure 3C and Table S5, Supporting Information). This metric is critical for assessing performance in 2PP applications, where high filler loadings can compromise resin transparency, viscosity, printability, and structural fidelity. A higher specific conductivity reflects more efficient charge transport per unit filler content, directly impacting device performance, material cost, and fabrication reliability. Metallic nanoparticles such as gold^[^ ^28^, ^59^, ^60^ ^]^ (Au, black squares) and silver^[^ ^61^, ^62^, ^63^ ^]^ (Ag, purple circles) exhibit exceptionally high specific conductivity values (≈10⁵ to 10⁶ S m^−1^ wt.%^−1^), but they are typically limited to planar (P) and low‐resolution structures due to particle aggregation and optical interference during laser exposure. Carbon‐based fillers such as carbon nanotubes^[^ ^25^, ^64^, ^65^ ^]^ (CNTs, blue up triangles) and graphene oxide^[^ ^29^, ^66^ ^]^ (GO, green down triangles) are more amenable to 3D printing but they exhibit lower specific conductivities (≈10^−^⁶–10^2^ S m^−1^ wt.%^−1^) because of dispersion challenges and restricted loading thresholds. Conducting polymers^[^ ^26^, ^67^, ^68^ ^]^ (CPs) have also been incorporated, often through post‐polymerization^[^ ^31^, ^69^ ^]^ (CP P.P., purple diamond) or post‐soaking^[^ ^34^, ^70^ ^]^ (CNT+CP P.S., green right triangle) techniques, achieving modest performance (≈10–10^3^ S m^−1^ wt.%^−1^). In the present study, we report a new formulation that synergistically combines MWCNTs (0.15 wt.%) and OE (0.4 wt.%) into a single homogeneous resin (CNT+OE, red star), achieving a specific conductivity exceeding 10⁵ S m^−1^ wt.%^−1^. This specific conductivity value (≈2.5 × 10^5^ S m^−1^ wt.%^−1^) surpasses all previously reported conductive filler‐based resins, while retaining structural resolution, uniformity, and optical clarity. This positions our formulation as the most efficient 2PP‐compatible conductive resin to date, combining high electrical performance and processability with unique advantages for microelectronic and optoelectronic applications (Figure 3C and Table S5, Supporting Information).

Laser scanning confocal microscopy was utilized to characterize the surface topography of the OEMWCNTMSs. As shown in the 3D view of color‐coded height map of surface texture (Figure 3D) and color‐coded height map of surface roughness (Figure 3E) structures fabricated from the composite resin containing 0.15 wt.% MWCNTs and 0.4 wt.% OE exhibited uniform surface texture and moderately rough surface, with an average roughness of ≈260 nm measured over 50 × 50 µm^2^ regions according to ISO 4287 using non‐contact confocal profilometry. This roughness is likely attributed to nanoscale heterogeneities introduced by MWCNT and OE dispersion yet remained within acceptable limits to preserve high‐resolution structural fidelity in 2PP‐fabricated features. Surface quality is a critical parameter for 2PP‐fabricated microstructures, particularly in bioelectronics and photonics, where nanoscale topology influences device performance and biological interactions. We evaluated the surface roughness (R_rms_) of OEMWCNTMSs using laser scanning confocal microscopy across a range of OE (0 to 0.4 wt.%) and MWCNT (0.05, 0.1, and 0.15 wt.%) concentrations (Figure 3F). The polymer microstructure (PEGDA only) exhibited a smooth surface with R_rms_ = 13 ± 1 nm. Addition of MWCNTs, without OE, increased roughness in a concentration‐dependent manner, with R_rms_ values of 30 ± 2 nm, 55 ± 2 nm, and 73 ± 3 nm for 0.05, 0.1, and 0.15 wt.% MWCNTs, respectively (*n* = 5, *p* < 0.001). This increase is presumably attributed to the inherent anisotropic shape and aggregation tendency of CNTs, which disrupt the smoothness of the cured matrix. Furthermore, incorporation of OE significantly modulated the surface topography. For each MWCNT concentration, increasing OE content led to a progressive increase in surface roughness. For instance, at 0.15 wt.% MWCNTs, R_rms_ increased from 73 ± 3 nm (0 wt.% OE) to 169 ± 5 nm (0.1 wt.% OE, *p* < 0.001), 196 ± 4 nm (0.2 wt.% OE, *p* < 0.001), 223 ± 3 nm (0.3 wt.% OE, *p* < 0.01), and 258 ± 2 nm (0.4 wt.% OE, *p* < 0.001). A similar statistically significant trend was observed for 0.05 and 0.1 wt.% MWCNT concentrations (*p* < 0.001 across groups). The observed increase in surface roughness is likely due to the phase separation and nanoscale aggregation of OE domains within the PEGDA matrix, which while beneficial for charge transport, contribute to nanostructural undulation. A correlation was observed between electrical conductivity and surface roughness (Figure 3A–F), indicating formulation‐dependent changes in filler distribution and crosslink density. Notably, incorporation of 0.4 wt.% OE and 0.15 MWCNTs increased conductivity to ≈10^5^ S m^−1^ while maintaining moderate surfaces roughness Rrms ≈ 260 nm). These results confirm that surface morphology can be systematically tuned by balancing CNT and OE content, offering design flexibility for applications requiring both conductivity and topographical control. Although conductivity enhancement slightly increases nanoscale roughness, the resin must achieve a high degree of homogeneity to remain printable; otherwise, the 2PP process becomes unstable. Thus, uniform resin mixing is a prerequisite for printability, whereas surface smoothness can be partially tuned through formulation optimization (Figure 3A–F). To assess reproducibility, surface roughness measurements were repeated on five independently fabricated samples for each composition (*n* = 5). All resin formulations exhibited an average Rrms with a coefficient of variation below 7%, indicating high reproducibility across fabrication batches (*p* > 0.05).

To demonstrate the potential application of OEMWCNTMSs in flexible microelectronics, we fabricated a micro‐printed circuit board (µPCB) (Figure 3G,H) comprising various electronic elements, including microresistors and microcapacitors, directly on a flexible PDMS substrate using the optimized conductive resin (0.15 wt.% MWCNTs and 0.4 wt.% OE). The straight‐line region in the I–V graph (Figure 3I) represents ohmic behavior of a resistor element (dimensions: 150 µm × 2 µm × 1 µm: length × width × height) located in the dashed region (Figure 3G), with a measured conductance of 61.4 ± 2.1 µS (*n* = 5). Figure 3J shows the I–V hysteresis loop (scan rate: 2 V s^−1^) for a microcapacitor (dimensions: 40 µm × 2 µm × 1 µm: length × width × height), highlighted in the red rectangle (Figure 3G). The rectangular shape of the I–V curve confirms capacitive behavior, with a specific capacitance of C_s_ = 667 ± 12 F g^−1^ at the scan rate 2 V S^−1^ (Equation S2, Supporting Information). The nearly rectangular I–V loops originate from reversible dielectric polarization and electronic charge accumulation within the composite rather than from faradaic or ionic processes. The polymer matrix alone showed a very small specific capacitance of C_s_ ≈ 27 µF g^−1^, consistent with its insulating nature. Adding 0.15 wt.% MWCNTs increased the specific capacitance to C_s_ ≈ 170 F g^−1^ through interfacial polarization and electronic conduction. The OE‐containing composite (0.4 wt.%) reached C_s_ ≈ 400 owing to enhanced electronic polarization within the conjugated polymer domains. The optimized OEMWCNTMSs (0.4 wt.% OE and 0.15 wt.% MWCNTs) exhibited the highest specific capacitance of ≈667, demonstrating a synergistic contribution from both conductive components. These results highlight the material's potential for use in solid‐state microcapacitors or dielectric energy‐storage elements where conformal, microscale patterning and mechanical flexibility are essential. The enhanced energy performance observed in the µPCB microcapacitors arises not only from the high conductivity of the dual‐filler composite but also from the 3D electrode geometry produced by 2PP. The printed interdigitated and volumetric microelectrode structures possess significantly larger effective surface area than planar thin‐film electrodes of equivalent footprint, facilitating greater charge accumulation at the electrode–electrolyte interface. Such 3D surface‐area effects have been widely reported to improve both areal and volumetric capacitance in microsupercapacitors fabricated by laser or direct‐writing methods.^[^ ^71^, ^72^ ^]^ Beyond miniaturization, the true 3D control offered by 2PP enables out‐of‐plane microelectrodes for volumetric tissues and curved substrates, monolithic vertical interconnects, and high‐surface‐area electrode architectures that enhance capacitance and reduce impedance. These capabilities are essential for next‐generation neural probes,^[^ ^73^ ^]^ biosensors,^[^ ^74^ ^]^ and micro‐energy devices,^[^ ^75^ ^]^ where volumetric current distribution and mechanical compliance cannot be achieved by conventional 2D fabrication.

**Figure 4:**
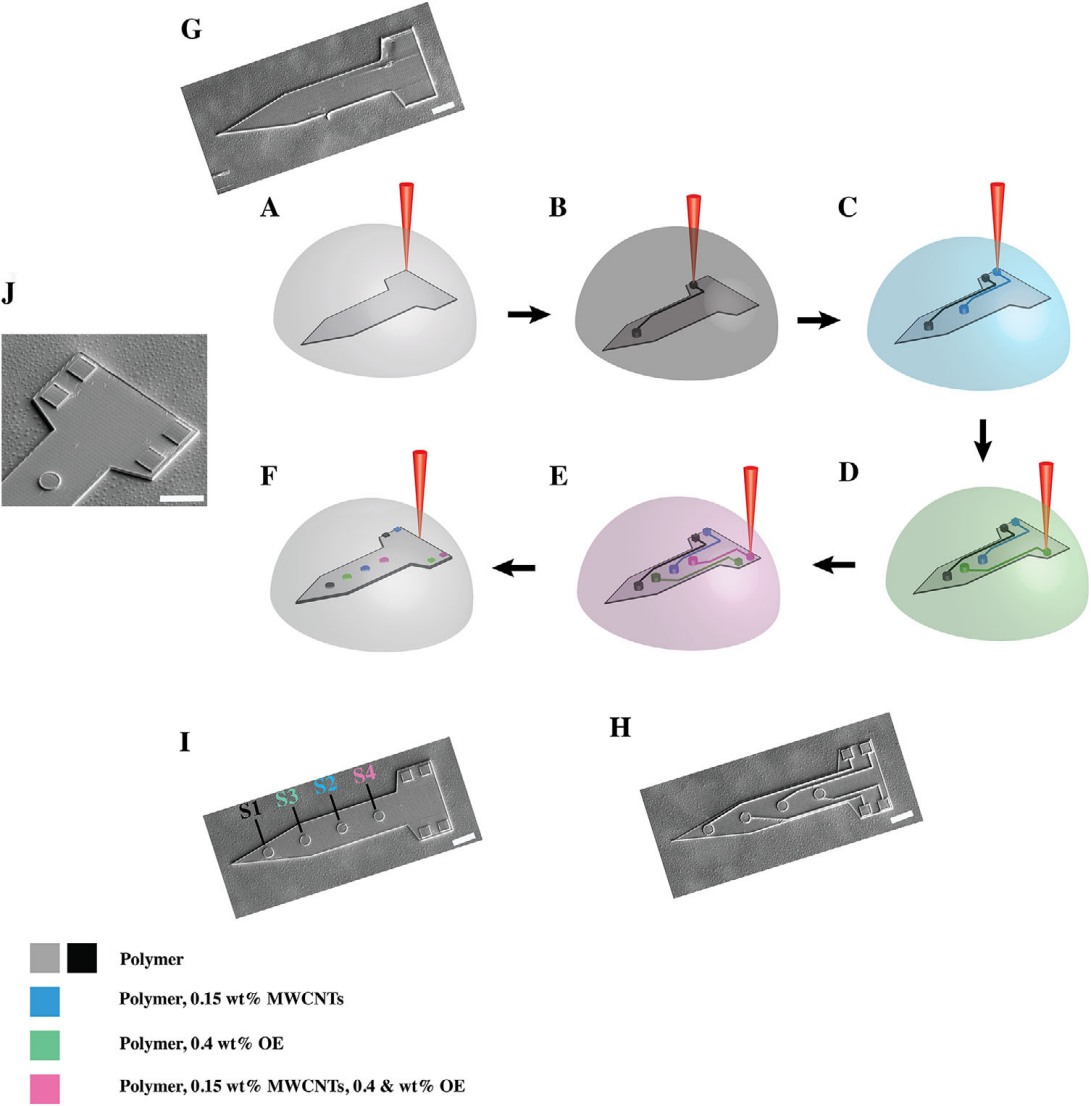
Multimaterial 2PP fabrication of composite microelectronic structures using OEMWCNTMSs. (A) SEM image of the base substrate patterned from PEGDA resin without conductive fillers. (A–F) Schematic overview of sequential fabrication steps for multimaterial electrode using four different resin formulations: (A) non‐conductive PEGDA structural base (gray), (B–F) subsequently, interconnect lines, recording sites, and contact pads were fabricated using four distinct ink formulations: (B) non‐conductive PEGDA (black), (C) PEGDA with 0.15 wt.% MWCNTs (blue), (D) PEGDA with 0.4 wt.% OE (green), and (E) PEGDA with both 0.15 wt.% MWCNTs and 0.4 wt.% OE (pink). (F) Illustration of partial encapsulation by non‐conductive PEGDA insulation layer, leaving electrode sites and recording pads exposed (gray). (G) SEM image of structural base. (H–J) SEM images of fully fabricated multimaterial microelectrodes incorporating all four resin types before (H) and after (I and J) insulation layer addition, confirming the geometric fidelity and clean material transitions between layers.

Traditional methods such as photolithography, sputtering, metal lift‐off, and etching are inherently limited by their planar nature, material restrictions, and need for complex, multi‐step processes involving alignment, masking, and post‐deposition treatments. These challenges hinder rapid prototyping, integration of dissimilar materials, and freeform geometries required for soft electronics and in vivo biointerfaces. To overcome these limitations, we introduce a multimaterial 3D microfabrication strategy based on sequential 2PP lithography, enabling spatially defined patterning of functionally distinct materials within a monolithic structure (**Figure** **4**). It is noteworthy that the feature size (line width) of ≈700 nm was achieved when a 1.7 mW femtosecond laser was focused by an oil‐immersion objective lens (63×, NA 1.4) and the composite resin was scanned at 50 µm s^−1^ (Figure S7, Supporting Information). Our fabrication sequence begins with the printing of a structural base using polymer resin without conductive fillers (gray, Figure 4A). Subsequently, interconnect lines, electrode sites, and contact pads were fabricated using four distinct resin formulations: i) polymer alone (black, Figure 4B); ii) polymer resin with 0.15 wt.% MWCNTs (blue, Figure 4C); iii) polymer resin with 0.4 wt.% OE (green, Figure 4D); and iv) polymer resin with 0.15 wt.% MWCNTs and 0.4 wt.% OE (pink, Figure 4E). The interconnects were designed with microscale dimensions of 2 µm height × 1 µm width, while electrode sites (S1, S2, S3, S4 in Figure 4I) were 20 µm diameter and 7 µm height cylinders, and square contact pads measured 20 × 20 × 7 µm^3^. A final insulating polymer layer was printed to selectively encapsulate the interconnects, leaving only the recording sites and contact pads exposed for direct functional access (Figure 4F). High‐resolution SEM images (Figure 4G–J) confirm the geometric fidelity and clean material transitions between layers. This approach demonstrates the capability to seamlessly fabricate multimaterial devices within one fabrication workflow and with heterogeneous electrical properties, rather than assembling separate elements post‐fabrication or transfer steps, resulting in a monolithic integrated device. The resultant multi‐site microelectrode contains electrode sites composed of different materials within one construct, enabling controlled, side‐by‐side evaluation of electrochemical and physiochemical studies under identical environmental and fabrication conditions. This multimaterial 2PP approach holds significant promise for a wide range of applications. In neural engineering, for example, distinct electrode formulations could be used to balance impedance, charge injection capacity, and long‐term stability across different sites. In microfluidics or soft robotics, embedded sensors and circuits with spatially varying mechanical and electrical responses can be printed within elastomeric bodies. Moreover, the ability to digitally assign materials within 3D volumes opens the door to customizable, closed‐loop microsystems with embedded logic, sensing, and actuation, bridging the gap between high‐resolution 3D printing and functional microelectronic integration.

**Figure 5:**
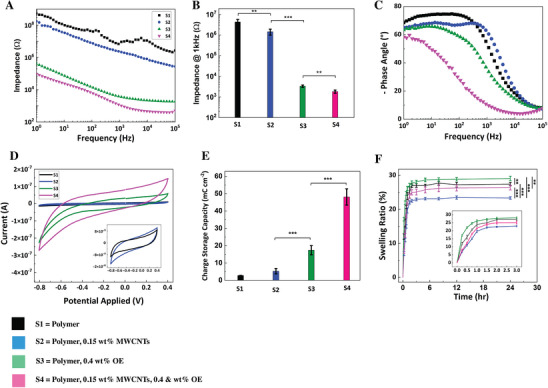
Electrochemical and swelling characterization of 2PP‐fabricated multimaterial microelectrodes. (A) Electrochemical impedance spectroscopy (EIS) profiles show impedance as a function of frequency for four resin formulations: S1 (PEGDA, black squares), S2 (PEGDA + 0.15 wt.% MWCNTs, blue circles), S3 (PEGDA + 0.4 wt.% OE, green triangles), and S4 (PEGDA + 0.15 wt.% MWCNTs + 0.4 wt.% OE, pink inverted triangles). (B) Bar graph of impedance at 1 kHz, illustrating significant reductions in S2–S4 compared to S1 (*n* = 5, *p* < 0.01, *p* < 0.001). (C) Phase angle as a function of frequency. S1 and S2 display capacitive behavior, while S3 and S4 exhibit a transition toward resistive behavior at higher frequencies, indicative of mixed ionic‐electronic conductivity. (D) Cyclic voltammetry (CV) curves at 0.1 V s^−1^ show increasing hysteresis loop area from S1 to S4, corresponding to enhanced redox activity. Inset: zoomed view of S1 and S2 for comparison. (E) Charge storage capacity (CSC), calculated from CV curves, increased significantly from 2.5 ± 0.6 mC cm^−^^2^ (S1) to 50.7 ± 4.5 mC cm^−^^2^ (S4) (*n* = 5, *p* < 0.001). (F) Swelling ratio (%) as a function of incubation time in PBS at room temperature. PEGDA hydrogel (S1) showed moderate swelling (≈27%), while CNT incorporation (S2) reduced it to ≈24%. OE (S3) increased swelling (≈29%) due to its hydrophilicity, and the composite formulation (S4) showed intermediate behavior (≈27%). Inset: magnified swelling profile over the first 3 h. Data shown as mean ± S.E.M, (*n* = 5). Statistical significance denoted as *p* < 0.01, *p* < 0.001.

The multimaterial microelectrode shown in Figure 4 was utilized to assess and characterize electrochemical and physiochemical properties of composite microstructures fabricated based on various formulations of photosensitive resin. To further evaluate electrochemical performance, we compared the electrical and physicochemical properties of four distinct electrode formulations printed via 2PP: S1 (polymer alone, black), S2 (polymer with 0.15 wt.% MWCNTs, blue), S3 (polymer with 0.4 wt.% OE, green), and S4 (polymer with 0.15 wt.% MWCNTs and 0.4 wt.% OE, pink) (Figures 4 and **5**). Electrochemical impedance spectroscopy (EIS) (**Figure** 5A) demonstrated that S1 exhibited the highest impedance across all frequencies, confirming its insulating nature. Incorporating 0.15 wt.% MWCNTs (S2) significantly reduced impedance due to formation of conductive pathways. The addition of 0.4 wt.% OE (S3) led to a further dramatic reduction in impedance, attributed to OE mixed ionic‐electronic conduction. The lowest impedance was observed in the dual‐filler composite 0.15 wt.% MWCNTs and 0.4 wt.% OE (S4), which maintained a consistent decrease across all frequencies, indicating synergistic charge transport enhancement as illustrated in Figure 3B. At the biologically relevant frequency of 1 kHz, impedance decreased significantly from 4.3 × 10⁶ ± 1.3 × 10⁶ Ω (S1, polymer alone) to 1.4 × 10⁶ ± 4.6 × 10⁵ Ω (S2, polymer with 0.15 wt.% MWCNTs, *p* < 0.01), 3.3 × 10^3^ ± 4.2 × 10^2^ Ω (S3, polymer with 0.4 wt.% OE, *p* < 0.001), and 1.8 × 10^3^ ± 3.2 × 10^2^ Ω (S4, polymer with 0.15 wt.% MWCNTs and 0.4 wt.% OE, *p* < 0.01) (Figure 5B).

The corresponding phase angle data (Figure 5C) further elucidated the electrochemical behavior. All sites showed capacitive characteristics at low frequencies (1–10 Hz), driven by double‐layer capacitance. S1 and S2 remained dominantly capacitive up to ≈10^3^ Hz, whereas S3 and S4 exhibited a sharp decrease in phase angle between 10 and 10^3^ Hz, indicating a transition from capacitive to resistive behavior due to enhanced Faradaic processes. Notably, S4 underwent this transition at ≈100 Hz and reached a nearly resistive phase (≈10°) at 1 kHz, suggesting dominant charge‐transfer interactions, which is consistent with hybrid ionic‐electronic conduction seen in OEMWCNT composite systems.^[^ ^54^, ^55^, ^56^ ^]^

Cyclic voltammetry (CV) was employed to probe redox and capacitive behavior across the four compositions (S1–S4) in the potential window −0.8 to +0.4 V at 0.1 V s^−1^ (Figure 5D). S1 (PEGDA) exhibited a flat profile, indicating negligible faradaic or capacitive response. S2 (MWCNTs + PEGDA) displayed a small, nearly rectangular loop characteristic of electric double‐layer capacitance (EDLC), with no distinct anodic or cathodic peaks, consistent with the typical rectangular CV signature of carbon‐based EDLC electrodes.^[^ ^76^ ^]^ S3 (OE + PEGDA) showed a larger loop with broad shoulders typical of polymer doping/de‐doping (pseudocapacitance) rather than discrete diffusion‐limited peaks, as reported for PEDOT:PSS (OE) films under comparable potential windows.^[^ ^77^ ^]^ S4, the composite of OE + MWCNTs + PEGDA, produced the largest and most rectangular loop, reflecting the superposition of MWCNT EDLC and PEDOT:PSS (OE) pseudocapacitance.^[^ ^78^ ^]^ Such carbon–polymer hybrids are known to exhibit complementary charge‐storage mechanisms, yielding higher total capacitance than either component alone.

The predominance of capacitive and pseudocapacitive processes at this scan rate explains the absence of sharp redox peaks: i) the MWCNT network contributes a nearly linear EDLC current across the potential window, ii) OE undergoes distributed, surface‐confined doping/dedoping that appears as broad shoulders, and iii) the conductive, porous composite network spreads effective redox potentials and overlaps EDLC currents, smoothing any localized faradaic response. Areal capacitance (C_A_) was calculated from the CV data (Equation S3, Supporting Information), yielding C_A_ = 0.246 ± 0.025 F cm^−^^2^ for S4, 0.145 ± 0.013 F cm^−^^2^ for S3, 0.016 ± 0.002 F cm^−^^2^ for S2, and 0.012 ± 0.007 F cm^−^^2^ for S1(PEGDA). These results confirm the synergistic contribution of EDLC of MWCNTs and pseudocapacitance of PEDOT:PSS, as the capacitance of the composite (S4) markedly exceeds the sum of its individual components, indicating efficient coupling between EDLC and pseudocapacitive mechanisms.^[^ ^79^, ^80^ ^]^ Similar synergistic enhancements have been widely reported for carbon/CP hybrids, including PEDOT:PSS–MWCNT electrodes and related polymer–carbon systems, wherein EDLC from the porous carbon network couples with polymer pseudocapacitance to yield capacitance exceeding either constituent alone.^[^ ^80^ ^]^ Consistent with capacitive/pseudocapacitive behavior, the first derivative (dI/dV) of the CV shows a smooth, low baseline without narrow extrema, derivative processing, which ordinarily amplifies discrete redox peaks, reveals no sharp features here, while any abrupt changes appear only at the scan reversals (Figure S10, Supporting Information). This aligns with the (quasi)‐rectangular voltammograms expected for EDLC/pseudocapacitive electrodes rather than diffusion‐limited redox systems.^[^ ^81^ ^]^ The potential‐switching response can be approximated by a diffusion‐limited time constant, $\tau \approx L_{\mathrm{eff}}^{2}/D_{\mathrm{ion}}$, as established for mixed ionic–electronic conductors.^[^ ^82^ ^]^ Here we take *L*_eff_to be the active PEDOT:PSS charging depth, on the order of our printed cylinder height 7 µm, consistent with thickness‐dependent transients in PEDOT:PSS devices, and we use literature *D*_ion_values for hydrated PEDOT:PSS in the range 10^−10^–10^−9^ m^2^ s^−11^ (the effective ionic diffusion coefficient).^[^ ^83^, ^84^, ^85^ ^]^ The expected electrochemical response time is τ ≈ 4–40 ms. This timescale, corresponding to a bandwidth of several tens of hertz, aligns with the quasi‐rectangular CVs recorded at 0.1 V s^−1^ and supports rapid, reversible doping/dedoping suitable for soft electronic applications.

Charge storage capacity (CSC) (derived from the CV curves, Equation S3, Supporting Information) increased with each functionalization step in polymer resin. CSC values were 2.8 ± 0.1 mC cm^−^^2^, 5.3 ± 1.6 mC cm^−^^2^, 17.4 ± 2.6 mC cm^−^^2^, and 48.1 ± 4.7 mC cm^−^^2^, for S1, S2, S3, and S4, respectively with statistically significant differences across all groups (*n* = 5, *p* < 0.01 and *p* < 0.001) (Figure 5E). These results highlight a ≈20‐fold increase in CSC from S1 to S4, consistent with prior findings that OE and CNT composites facilitate enhanced charge accumulation and transport due to their synergistic interaction.^[^ ^56^, ^86^ ^]^ Together, these findings validate the superior electrochemical behavior of the S4 formulation, confirming its high potential for microscale applications requiring low impedance, enhanced charge transfer, and efficient capacitive energy storage. The 3D architecture provides an enlarged effective surface area compared with planar analogues, directly contributing to enhanced capacitance and lower impedance.

To evaluate the swelling behavior of multimaterial microstructures, we monitored volumetric expansion in PBS over 24 h (Figure 5F). All samples exhibited rapid initial swelling within the first 3 h (inset), followed by stabilization. The PEGDA‐only formulation (S1, black) reached a final equilibrium swelling ratio of 27.5 ± 0.5%. Incorporation of 0.15 wt.% MWCNTs (S2, blue) resulted in a reduced swelling ratio of 23.3 ± 0.4%, representing a significant ≈10% decrease relative to S1 (*n* = 5, *p* < 0.001). This reduction can be attributed to CNT‐induced densification of the hydrogel network and increased physical entanglement, which constrain polymer chain mobility and water uptake. Such effects are in line with previous studies reporting that CNTs lower hydrogel swelling by increasing effective crosslink density and limiting osmotic expansion.^[^ ^87^ ^]^ In contrast, the addition of 0.4 wt.% OE to the PEGDA matrix (S3, green) led to a significant increase in swelling ratio to 29.1 ± 0.7% (*n* = 5, *p* < 0.001). This ≈7% enhancement relative to PEGDA alone reflects the hydrophilic nature of OE, particularly its polystyrene sulfonate component, which introduces ionic functional groups that elevate water affinity. Furthermore, microphase separation between OE‐rich and PEGDA‐rich domains has been shown to facilitate localized osmotic gradients, thereby promoting swelling.^[^ ^88^ ^]^ Interestingly, the dual‐filler formulation (S4, pink), containing both 0.15 wt.% MWCNTs and 0.4 wt.% OE, produced an intermediate swelling ratio of 26.4 ± 0.7%, statistically distinguishable from both S2 and S3 (*n* = 5, *p* < 0.01), illustrating a balance between the swelling‐reducing effect of CNTs and the swelling‐promoting nature of OE. Collectively, these results highlight the complex interplay between nanoscale fillers within a hydrogel matrix and underscore the need for compositional tuning to optimize functional properties, which is an important consideration for designing hydrogels with tailored dimensional stability in biological and microfluidic environments.

To assess the long‐term structural stability of the OEMWCNTMSs in aqueous conditions, we monitored the mass loss of each formulation over a 10‐day period (Figure S11, Supporting Information). All samples exhibited minimal degradation, with total mass loss remaining below 6% across the observation window. The PEGDA‐only sample (S1, black) showed a gradual increase in mass loss, reaching ≈7.3 ± 0.8% by day 10. Incorporation of MWCNTs (S2, blue) and OE (S3, green) slightly reduced the rate and extent of degradation, with final mass losses of 5.8 ±0.9% and 5.3 ± 0.6%, respectively. The composite formulation containing both MWCNTs and OE (S4, pink) exhibited the lowest cumulative mass loss of 3.5 ± 0.3% by day 10. Importantly, statistical analysis revealed no significant differences in mass loss between groups (*n* = 5, *p* > 0.05), indicating that incorporation of conductive fillers did not adversely affect the hydrolytic stability of the PEGDA matrix. These results suggest that all composite formulations retain their structural integrity over time and are suitable for aqueous bioelectronic applications requiring durability and mechanical robustness.

To further confirm electrochemical durability, we assessed impedance stability of the S4 formulation following incubation in PBS for 1, 2, and 3 days (Figure S12, Supporting Information). Across the full frequency range (1 Hz–100 kHz), impedance curves showed minimal deviation. For instance, at 1 kHz, impedance increased modestly to a total relative change of ≈9%. This stability correlates well with the low mass loss observed over 10 days, confirming that the dual‐filler PEGDA‐based composite maintains both structural integrity and electrochemical functionality in aqueous solution.

**Figure 6:**
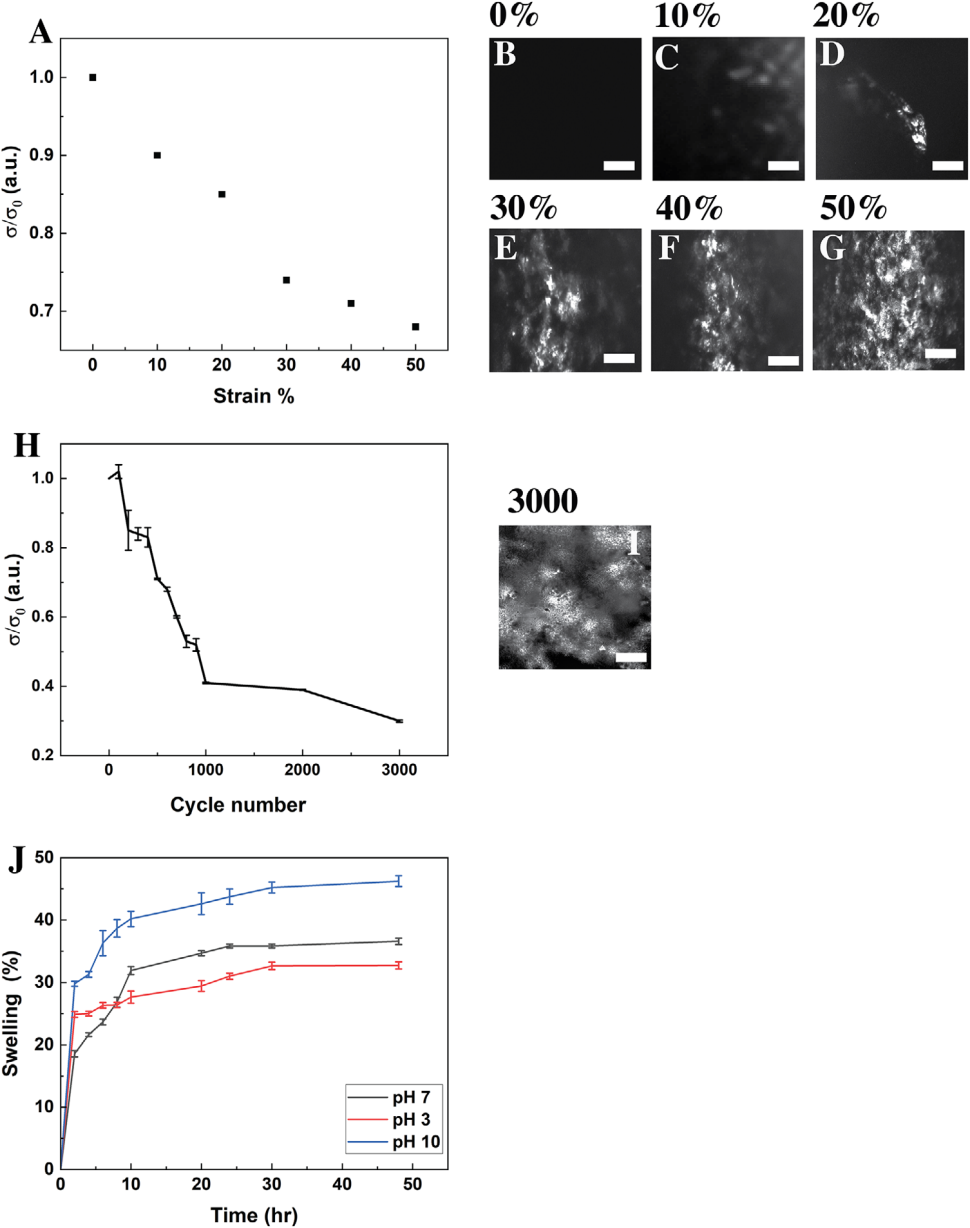
Electro‐mechanical durability and pH stability of 2PP‐printed OEMWCNTs composites on PDMS. (A) Normalized conductivity (σ/σ_0_) as a function of applied tensile strain (10–50%). The composite retained ≈ 65% of its initial conductivity at 50% strain; testing beyond this point was limited by PDMS substrate failure. (B–G) In situ optical micrographs at 0%, 10%, 20%, 30%, 40%, and 50% strain showing progressively brightened crack patterns corresponding to localized fracture of the conductive network (scale bar = 500 µm). (H) Normalized conductivity (σ/σ_0_) during cyclic stretching at 10% strain and 10 Hz for up to 3000 cycles (mean ± SD, *n* = 3, p < 0.01). (I) Optical micrograph after 3000 cycles showing distributed crack patterns without delamination (scale bar = 500 µm). (J) Swelling ratio of the optimized composite in PBS (37 °C) at pH 3, 7, and 10 over 48 h (mean ± SD, *n* = 5, p < 0.01), demonstrating modest pH‐dependent swelling and excellent structural integrity.

To quantify strain tolerance, we measured the normalized conductivity (σ/σ_0_)of PDMS‐supported OEMWCNTs composites (0.4 wt.% PEDOT:PSS + 0.15 wt.% MWCNTs) under monotonic tensile strain (**Figure** **6**A). Conductivity decreased progressively from 10% to 50% strain, with the composite retaining ≈ 65% of its initial conductivity at 50% strain; testing beyond this point was limited by substrate failure. In situ optical microscopy at each strain level (Figure 6B–G) revealed the progressive appearance of bright regions corresponding to microcracks within the composite, but no delamination or detachment from the PDMS substrate (Figure S13A,B, Supporting Information). These bright features arise from enhanced light scattering and reflection at fractured, indicating fragmentation of the conductive network consistent with the observed drop in normalized conductivity (σ/σ₀). Cyclic durability was then evaluated at 10 % uniaxial strain and 10 Hz for up to 3000 cycles. The composite retained ≈86% of its initial conductivity after 400 cycles, ≈40% after 1000 cycles, and ≈30% after 3000 cycles (Figure 6H). Post‐cycling optical images (Figure 6I) displayed fine surface cracking but no delamination or detachment from the PDMS substrate (Figure S13C, Supporting Information). The gradual conductivity decrease is attributed to microstructural fatigue within the PEDOT:PSS–MWCNT percolation network and localized debonding at filler–matrix interfaces, which increase inter‐domain tunneling resistance during repeated deformation. Despite these losses, electrical continuity and adhesion were preserved, demonstrating mechanical compliance and partial self‐recovery of the conductive network. Complementary bending and flexion tests (2 µm, 500 µm, 1 mm, and 2 mm deflection at 25 µm s^−1^ for 500 cycles) similarly showed no delamination (Figure S13D–H, Supporting Information).

To assess environmental stability, swelling studies were conducted on the optimized composite (S4) in PBS (37 °C) at pH 3, 7, and 10 for 48 h. Following the approach of Park and co‐workers,^[^ ^89^ ^]^ we examined the structural response under varying pH conditions. As shown in Figure 6J, the equilibrium swelling ratio increased moderately from ≈33% at pH 3 to ≈46% at pH 10, reflecting enhanced ionization of sulfonate groups within the PSS phase at basic pH. Despite this modest change, the composite maintained its geometry and adhesion without visible cracking or delamination, confirming excellent chemical and mechanical robustness. These results align with prior pH‐robustness evaluations and further support the material's suitability for bioelectronic and soft‐device applications operating across diverse environments.^[^ ^89^ ^]^

## 3 Conclusion

This work advances two‐photon polymerization (2PP) for additive manufacturing of soft electronics by developing a multimaterial resin system that integrates organic electronic components and carbon nanotube fillers. The resulting hybrid resin achieves a rare combination of electrical conductivity, optical transparency, and structural resolution, addressing long‐standing limitations of light‐mediated microscale fabrication. Using this approach, we directly fabricated functional microresistors and microcapacitors on flexible substrates, demonstrating digital patterning of conductive, insulating, and electroactive domains within a single 3D microdevice—without post‐fabrication processing.

Comprehensive characterization revealed capacitive and pseudocapacitive charge storage arising from synergistic coupling between PEDOT:PSS and MWCNTs, along with robust electro‐mechanical performance under cyclic stretching (10% strain, 3000 cycles) and strong adhesion to PDMS. The material also maintained structural integrity and modest swelling across a broad pH range (3–10), confirming its chemical and mechanical stability under physiologically relevant conditions. Together, these results establish a versatile 2PP platform for fabricating functional soft microelectronics with tunable electrochemical behavior and high geometric precision. The demonstrated compatibility of the resin with flexible substrates and its stability under deformation and environmental stress suggest promising applicability in biomedical interfaces, micro‐energy storage, and wearable devices. Future research may focus on integrating biological or sensing functionalities within the resin matrix to enable next‐generation adaptive biointerfaces and responsive microelectronic systems fabricated entirely by 2PP

## 4 Experimental Section

### Materials

Poly(ethylene glycol) diacrylate (PEGDA, *M_n_* = 700), high‐conductivity grade poly(3,4‐ethylenedioxythiophene):poly(styrenesulfonate) (PEDOT:PSS, 1.0 wt.% in H_2_O), 3‐(trimethoxysilyl)propyl methacrylate, and pentaerythritol tetrakis(3‐mercaptopropionate) (PETMP) were obtained from Sigma‐Aldrich. Dimethyl sulfoxide (DMSO, molecular biology grade), phosphate‐buffered saline (PBS) tablets (for 100 mL, biotechnology grade), ethanol (200 proof), and Sylgard™ 184 Silicone Elastomer Kit were purchased from VWR. Acid‐functionalized multi‐walled carbon nanotubes (MWCNTs; purity >95 wt.%, outer diameter <8 nm, length 10–30 µm, COOH content ≈3.9 wt.%) were supplied by Cheap Tubes Inc. Ethyl (2,4,6‐trimethylbenzoyl)phenylphosphinate (TPO‐L) was obtained from Oakwood Chemical.

### Resin Preparation

The base resin formulation was prepared by dispersing multi‐walled carbon nanotubes (MWCNTs) in a mixture of pentaerythritol tetrakis(3‐mercaptopropionate) (PETMP), ethyl (2,4,6‐trimethylbenzoyl)phenylphosphinate (TPO‐L), and poly(ethylene glycol) diacrylate (PEGDA). The suspension was magnetically stirred for 10–12 h to achieve uniform dispersion of MWCNTs, followed by centrifugation at 4700 rpm for 30 min (Allegra X‐30R, Beckman Coulter) to remove large agglomerates. Subsequently, dimethyl sulfoxide (DMSO) and poly(3,4‐ethylenedioxythiophene):poly(styrenesulfonate) (PEDOT:PSS, OE) were added, and the mixture was stirred for an additional 1 h. The final resin formulation contained 0–0.4 wt.% OE (i.e., PEDOT:PSS), 24.6–25 wt.% DMSO, 0–0.15 wt.% MWCNTs, 18.75 wt.% PETMP, 2 wt.% TPO‐L, and the remainder PEGDA. A control resin composed of PEGDA and TPO‐L only was prepared for comparison.

### Design of Microstructures

3D microstructures were designed using Autodesk Fusion 360 and exported in stereolithography (STL) format. The CAD files were then imported into nFab software (version 5.0.14, Newport) for slicing, path generation, and subsequent execution of two‐photon polymerization (2PP) fabrication.

### Two‐Photon Lithography

A droplet of homogenized resin was deposited between a standard glass slide and either a silanized coverslip or a silanized PDMS film. The assembly was mounted on a piezoelectric Z‐stage (VP‐5ZA, Newport) integrated with high‐precision X–Y translation stages (XMS160, Newport). A 63× oil‐immersion objective (Plan N, Olympus) was used to focus the laser at the resin–substrate interface. Two‐photon polymerization was initiated using 130 fs laser pulses from a Ti:sapphire oscillator (Mai Tai™ DeepSee, Spectra‐Physics) operating at 80 MHz, centered at 780 nm, with an average laser power of 1.7 mW at the objective focal plane. The tightly focused beam induced localized crosslinking of the resin, enabling layer‐by‐layer fabrication of microstructures in an inverted configuration on the coverslip or PDMS substrate. The stage motion was controlled through a computer‐interfaced motion controller and driver system (XPS, Newport) in conjunction with nFab software (v5.0.14, Newport), using a writing speed of 50 µm s^−1^. After printing, the substrate was gently rinsed with ethanol for 30 s to remove unpolymerized resin and air‐dried before further analysis.

### Surface Treatment

To promote adhesion of 2PP‐fabricated microstructures to glass coverslips or PDMS substrates, surface silanization was performed using a methacrylate‐based coupling agent. The silanization solution consisted of 100 µL of 3‐(trimethoxysilyl)propyl methacrylate, 2 mL of ethanol, and 6 mL of 10% (v/v) aqueous acetic acid. A volume of 200 µL of this solution was applied onto each substrate and allowed to react for 5 min at room temperature. Following incubation, the excess solution was removed, and the surface was gently rinsed with ethanol and air‐dried prior to resin deposition.

### PDMS Molding

The base elastomer and curing agent (Sylgard™ 184, Dow Corning) were mixed at a 10:1 (w/w) ratio and degassed under vacuum for 1 h to remove trapped air bubbles. The mixture was then poured into a glass mold and spin‐coated at 100 rpm for 5 s to achieve a uniform film thickness. Subsequently, the PDMS was cured at 60 °C for 2 h in a convection oven. After curing, the flexible PDMS sheets (≈200 µm thick) were carefully peeled from the glass substrate using tweezers and stored in a dust‐free environment prior to use.

### UV–Vis Spectroscopy

The optical transparency of the photosensitive resins was characterized using UV–Vis spectroscopy (SpectraMax M5, Molecular Devices). Resin formulations with varying compositions were dispensed into 3 mL quartz cuvettes (PerkinElmer Instruments), and transmittance spectra were recorded over the 350–750 nm wavelength range in 10 nm intervals. An empty cuvette was used as a reference to normalize the transmittance data.

### Partially Gold‐Coated Substrate

Glass coverslips were selectively masked with temperature‐resistant tape to define deposition regions. A 10 nm chromium adhesion layer followed by a 100 nm gold layer was sequentially deposited onto the exposed areas using electron‐beam evaporation (Thermionics eBeam Evaporator, Thermionics). After deposition, the masking tape was carefully removed, yielding partially gold‐coated substrates suitable for patterned microfabrication and electrical interfacing.

### Electrical Property

Current–voltage (I–V) characteristics were measured using a semiconductor parameter analyzer (B1500A, Keysight Technologies). Probe tips with a 1 µm diameter (Signatone), connected to the Source Measure Unit (SMU‐8, Keysight), were positioned at both ends of the printed microstructure under a stereo optical microscope (Discovery V8, ZEISS, Germany) to ensure precise electrical contact. A voltage sweep from −3 to +3 V was applied in 50 mV increments, and the corresponding current response was recorded. For capacitance measurements, cyclic hysteresis loops were obtained over the −3 to +3 V potential range at a scan rate of 2 V s^−1^. All data were processed using EasyEXPERT group+ software (Keysight Technologies). Electrical conductance was extracted from the linear slope of the I–V curve, and conductivity was calculated from the microstructure's geometric dimensions.

### Optical Microscopy

Brightfield optical images of the fabricated microstructures were captured using an upright microscope (Imager Z1, ZEISS, Germany) and a stereomicroscope (Discovery V8, ZEISS, Germany). The upright microscope was used for high‐magnification imaging to evaluate structural fidelity and surface morphology, whereas the stereomicroscope enabled low‐magnification inspection of larger‐area features and electrode alignment during electrical measurements. Image acquisition and processing were performed using ZEN‐pro and Axiovision software (ZEISS, Germany) to assess structural integrity, resolution, and feature uniformity. For enhanced visual clarity, the contrast and brightness were digitally adjusted using Adobe Photoshop. These adjustments were applied uniformly across all images and did not alter the morphology or features.

### Scanning Electron Microscopy

Microfabricated samples were mounted onto aluminum stubs using double‐sided carbon tape and sputter‐coated with a thin gold layer for 90 s at 40 mA using a desktop sputtering system (DESK‐II, Denton Vacuum LLC). Scanning electron micrographs were acquired with a field‐emission scanning electron microscope (XL‐30S FEG, FEI, PHILIPS) operated at an accelerating voltage of 3 kV in secondary electron detection mode. For enhanced visual clarity, the background region of the substrate, affected during sputter coating, was digitally adjusted using Adobe Photoshop. These modifications were applied uniformly across the image and did not alter the morphology or features of the printed 3D microstructures.

### Morphological Assessment

Surface texture and roughness profiles of the 2PP‐fabricated microstructures were characterized using non‐contact Laser Confocal Surafce Microscopy (LSM800, ZEISS, Germany) equipped with an Epiplan‐Apochromat 50× objective lens (NA 0.95, DIC). ConfoMap software (ZEISS, Germany) was employed for data processing in accordance with ISO 4287 standards. The MCM system generated 3D, color‐coded height maps representing the surface morphology and topography of the printed microstructures (Figure S9, Supporting Information). The precision motorized stage provided positional resolution down to 10 nm. Areal roughness measurements were conducted over a 50 µm × 50 µm scan area to capture complete topographic information beyond linear profiling. The root‐mean‐square roughness (R_aeal_ = Rrms) was reported as the primary surface roughness parameter. To evaluate reproducibility, measurements were repeated on five independently fabricated samples for each resin composition (*n* = 5). All resin formulations exhibited a mean Rrms with a coefficient of variation below 7%, indicating high reproducibility across fabrication batches (one‐way ANOVA, *p* > 0.05).

### Mixed Ion‐Electron Conductivity and Electrochemical Property

Mixed ion–electron transport behavior was evaluated using electrochemical impedance spectroscopy (EIS) and cyclic voltammetry (CV), performed on an Autolab PGSTAT302N potentiostat (Metrohm, USA) controlled by Nova software (v2.1). Measurements were carried out in a standard three‐electrode configuration, consisting of a platinum wire counter electrode, an Ag/AgCl reference electrode, and the fabricated microelectrode as the working electrode, all immersed in phosphate‐buffered saline (PBS, pH 7.4). For EIS, a 10 mV sinusoidal voltage perturbation was applied across a frequency range of 1–10⁵ Hz to probe charge‐transfer and interfacial resistance characteristics. For CV, the working electrode potential was swept between –0.8 and +0.4 V versus Ag/AgCl at a scan rate of 0.1 V s^−1^ using the staircase method. The third cycle was used for analysis to ensure electrochemical stability and reproducibility. The charge storage capacity (CSC) was determined by integrating the enclosed area of the CV curve and normalizing to the scan rate. These analyses enabled quantitative evaluation of both electronic conduction through the PEDOT:PSS–MWCNT network and ionic transport within the hydrated polymer matrix, reflecting the composite's mixed conduction characteristics.

### Stretching Test

The fabricated test pattern was a rectangular strip (length: 2.5 cm; width: 3 mm; thickness: 2 mm) on silanized PDMS substrates (thickness 2 mm). Then, the structures were encapsulated under a thin PDMS sheet. Uniaxial cyclic stretching was performed using a custom‐built tensile apparatus, in which one end of the PDMS sample was fixed and the opposite end attached to a sub‐micron precision motorized X‐stage (XML50, Newport, USA). Samples were subjected to strain 10% at 25 µm s^−1^ with frequency 10 Hz for up to 3000 cycles. For monotonic strain analysis (10–50% strain**),** a new sample was used for each strain level to eliminate any prior deformation history and ensure accurate assessment of strain‐dependent conductivity.

### Bending Test

To evaluate flexural stability, printed films containing the same optimized formulation were fabricated as rectangular strips (length: 2.5 cm; width: 5 mm; thickness: 2 mm) and subsequently laminated under PDMS (thickness 2 mm). Mechanical stability under cyclic bending was tested using a custom‐built flexion apparatus, where each sample was suspended with the resin side facing downward above a motorized Z‐stage (GTS30V, Newport, USA). A spherical probe (diameter = 4 mm) mounted on the stage applied controlled downward deflections at a velocity of 25 µm s^−1^ with a frequency 10 Hz to simulate repeated flexion.

### Swelling and Degradation Studies

Following fabrication, the printed microstructures were immersed in ethanol for 2 h to remove unpolymerized residues. The initial mass (W₀) of each sample was measured using an analytical balance (XPR504S, Mettler Toledo). Each sample was then incubated at 37 °C in 1 mL of commercial PBS (pH 7.4), or PBS adjusted to pH 3 (HCl) or pH 10 (NaOH) for up to 48 h to assess swelling behavior under different pH conditions. At each designated time point, swollen samples (*n* = 5) were removed from PBS, gently blotted with Kimwipes to remove surface moisture, and weighed to obtain the swollen mass (Ww). Subsequently, the samples were dried in a vacuum oven and re‐weighed to determine the dry mass (Wd).

(1) $$Swellingratio\;\%=\frac{\left(W_{w}-W_{d}\right)}{W_{w}}\times 100$$

(2) $$Massloss\;\%=\frac{\left(W_{0}-W_{d}\right)}{W_{0}}\times 100$$

### Graphing and Statistical Analysis

Data visualization and statistical analysis were performed using OriginPro software (OriginLab, Northampton, MA). Statistical significance among sample groups was assessed using one‐way analysis of variance (ANOVA) followed by Tukey's post hoc test. All results are expressed as mean ± standard deviation (SD) or standard error of the mean (SEM), as indicated. Statistical significance was denoted as: *p < 0.01, **p < 0.001.

## Conflict of Interest

The authors declare no conflict of interest.

## Supporting information



Supporting Information

## Data Availability

The data that support the findings of this study are available from the corresponding author upon reasonable request.
