# Supplementary material for: Multimaterial 3D Printing of Soft and Stretchable Electronics
Source: Adv Sci (Weinh). 2025 Nov 21;13(8):e13208. doi: 10.1002/advs.202513208 (PMC12884721; doi:10.1002/advs.202513208)
Supplement: Supplementary file 1 — Supporting Information [file ADVS-13-e13208-s001.docx]

Supporting Information

Multimaterial 3D Printing of Soft and Stretchable Electronics

Omid Dadras-Toussi, Bhoomija Hariprasad, and Mohammad Reza Abidian*

Formulation of various photosensitive resins

MWCNTs required a minimum of 10 h (overnight) of magnetic stirring in PETMP to achieve a uniform dispersion in the resin. In contrast, OE dissolved readily after the addition of DMSO and typically reached full dispersion within 2 h; however, extended stirring promoted OE aggregation. When all components were mixed simultaneously, OE aggregated early and was removed during centrifugation before the MWCNTs had fully homogenized. To resolve this incompatibility, a sequential two-step mixing protocol was developed for preparing the composite conductive resin.

The compositions for photosensitive resins containing a single organic conductive filler are listed in Table S1 (MWCNT-based) and Table S2 (OE-based). Table S3 summarizes formulations incorporating both MWCNTs and OE at varying concentrations, and Table S4 presents the four primary formulations selected for comparative electrical and electrochemical characterization. All compositions are reported in weight percent (wt%) relative to the total resin mass. For consistency, each formulation was prepared as a 2 g batch, with component masses calculated accordingly. Across all formulations, PEGDA and TPO-L were fixed at 54.2 wt% and 1.995 wt%, respectively. The combined OE–DMSO content was maintained at 25 wt%, and the total MWCNT–PETMP fraction was held constant at 18.805 wt%.

**Table S1.** Formulation of photosensitive resin containing only MWCNTs.

| MWCNTs  (wt%) | OE  (wt%) | PETMP  (wt%) | DMSO (wt%) | PEGDA  (wt%) | TPO-L  (wt%) |
| --- | --- | --- | --- | --- | --- |
| 0 | 0 | 18.805 | 25 | 54.2 | 1.995 |
| 0.05 | 0 | 18.755 | 25 | 54.2 | 1.995 |
| 0.1 | 0 | 18.705 | 25 | 54.2 | 1.995 |
| 0.15 | 0 | 18.655 | 25 | 54.2 | 1.995 |

**Table S2.** Formulation of photosensitive resin containing only OE.

| MWCNTs  (wt%) | OE  (wt%) | PETMP  (wt%) | DMSO (wt%) | PEGDA  (wt%) | TPO-L  (wt%) |
| --- | --- | --- | --- | --- | --- |
| 0 | 0 | 18.805 | 25 | 54.2 | 1.995 |
| 0 | 0.1 | 18.805 | 24.9 | 54.2 | 1.995 |
| 0 | 0.2 | 18.805 | 24.8 | 54.2 | 1.995 |
| 0 | 0.3 | 18.805 | 24.7 | 54.2 | 1.995 |
| 0 | 0.4 | 18.805 | 24.6 | 54.2 | 1.995 |

**Table S3.** Formulation of composite resin with various MWCNTs and OE content.

| MWCNTs  (wt%) | OE  (wt%) | PETMP  (wt%) | DMSO (wt%) | PEGDA  (wt%) | TPO-L  (wt%) |
| --- | --- | --- | --- | --- | --- |
| 0.05 | 0.1 | 18.755 | 24.9 | 54.2 | 1.995 |
| 0.05 | 0.2 | 18.755 | 24.8 | 54.2 | 1.995 |
| 0.05 | 0.3 | 18.755 | 24.7 | 54.2 | 1.995 |
| 0.05 | 0.4 | 18.755 | 24.6 | 54.2 | 1.995 |
| 0.1 | 0.1 | 18.705 | 24.9 | 54.2 | 1.995 |
| 0.1 | 0.2 | 18.705 | 24.8 | 54.2 | 1.995 |
| 0.1 | 0.3 | 18.705 | 24.7 | 54.2 | 1.995 |
| 0.1 | 0.4 | 18.705 | 24.6 | 54.2 | 1.995 |
| 0.15 | 0.1 | 18.655 | 24.9 | 54.2 | 1.995 |
| 0.15 | 0.2 | 18.655 | 24.8 | 54.2 | 1.995 |
| 0.15 | 0.3 | 18.655 | 24.7 | 54.2 | 1.995 |
| 0.15 | 0.4 | 18.655 | 24.6 | 54.2 | 1.995 |

**Table S4.** Formulation of 4 main photosensitive resin

| Name | MWCNTs  (wt%) | OE  (wt%) | PETMP  (wt%) | DMSO (wt%) | PEGDA  (wt%) | TPO-L  (wt%) |
| --- | --- | --- | --- | --- | --- | --- |
| Polymer (without fillers) | 0 | 0 | 18.705 | 25 | 54.2 | 1.995 |
| MWCNTs | 0.15 | 0 | 18.655 | 25 | 54.2 | 1.995 |
| OE | 0 | 0.4 | 18.805 | 24.6 | 54.2 | 1.995 |
| MWCNTs & E | 0.15 | 0.4 | 18.655 | 24.6 | 54.2 | 1.995 |

**Resin Stability and Storage**

As shown in Figure S1, centrifugation of the resin containing 0.15 wt% MWCNTs effectively removed residual nanotube aggregates that would otherwise interfere with two-photon polymerization (2PP) fabrication. Although these aggregates are not discernible by the naked eye, they are readily observed under high-magnification objective lenses and must be eliminated to ensure consistent printing fidelity.


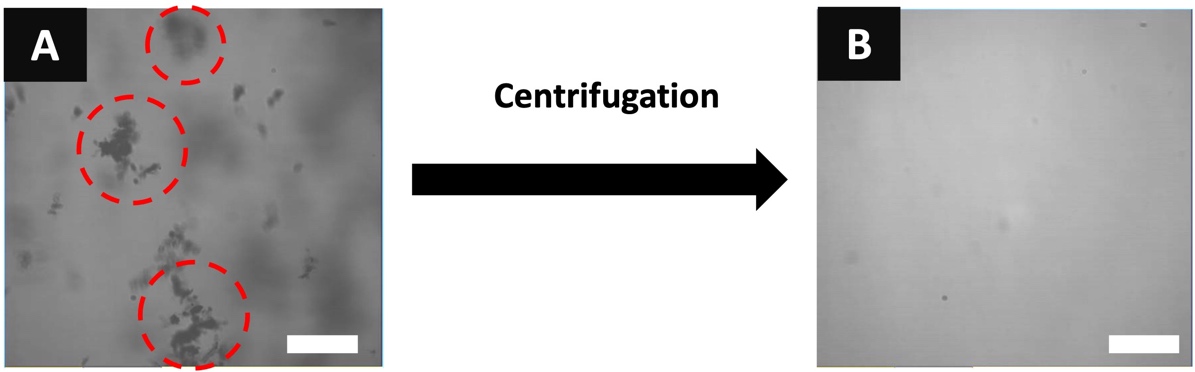


**Figure S1.** Optical micrographs of resin before (A) and after (B) centrifugation. Scale bar: 5 μm. Large MWCNTs aggregates are removed after centrifugation of the resin.

As shown in **Figure S2**, increasing the MWCNT concentration beyond 0.15 wt% did not yield a meaningful improvement in electrical conductivity. The measured conductivities of microstructures fabricated from MWCNT-only conductive resins at 0.15 wt%, 0.2 wt%, 0.3 wt%, and 0.4 wt% were 8 ± 1.4 S m⁻¹, 13 ± 2.8 S m⁻¹, 9 ± 2.2 S m⁻¹, and 12 ± 1.9 S m⁻¹, respectively. These values show no statistically significant differences, indicating that ~0.15 wt% represents the maximum effective dispersible concentration of MWCNTs in the resin. At higher nominal loadings, the excess nanotubes likely form aggregates that are removed during centrifugation, resulting in no net increase in the amount of conductive filler within the printable resin and thus no enhancement in conductivity.


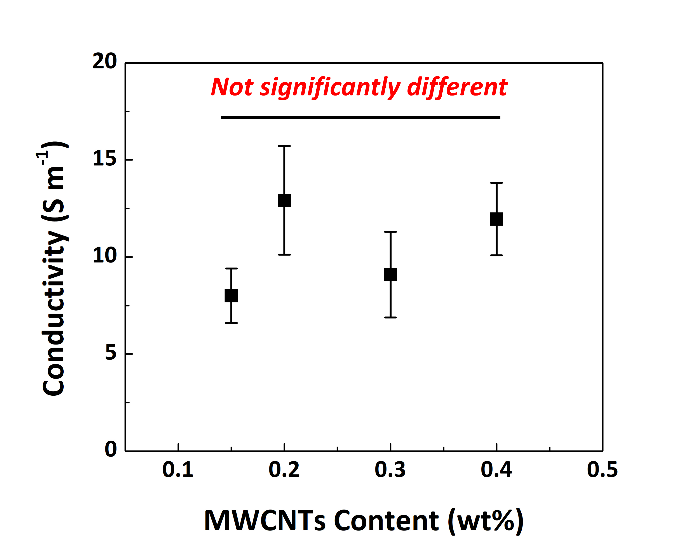
As shown in **Figure S3**, resins containing 0.4 wt% and 0.5 wt% OE were prepared and imaged using optical microscopy immediately after preparation. For the resin with 0.4 wt% OE, no visible aggregates were observed (**Figures S3A** and **S3C**), indicating a stable and homogeneous dispersion. In contrast, the resin containing 0.5 wt% OE exhibited pronounced aggregation (**Figures S3B** and **S3D**), clearly visible under optical microscopy. These findings demonstrate that exceeding the optimized concentration of 0.4 wt% OE results in immediate resin instability, rendering it unsuitable for lithography-based fabrication techniques.

**Figure S2** Conductivity of microbars as a function of concentration of MWCNTs (no OE).


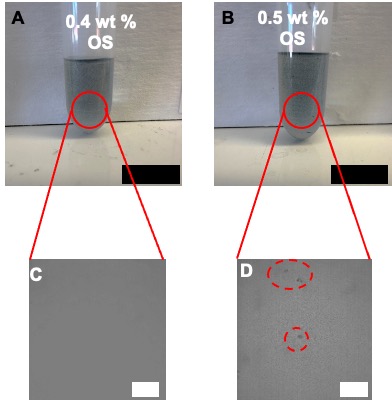


0.4 wt%

OE

0.5 wt%

OE

**Figure S3.** Optical assessment of resin homogeneity immediately after preparation. (A) Optical micrograph of resin containing 0.4 wt% OE, showing uniform dispersion without detectable aggregation. (B) Resin containing 0.5 wt% OE, in which visible aggregates begin to form. (C, D) Higher-magnification views corresponding to panels A and B, respectively. Red dotted circles highlight regions of OE aggregation. Scale bars: 1 mm.

The optimized dual-filler resin remained stable and fully printable for ≈ 6 h at room temperature (25 °C), consistent with the conditions used for 2PP fabrication (**Figures S4A–S4F**). Resin samples were protected from light between time points to prevent photoinitiated polymerization due to photosensitivity. For long-term storage, the resin can be preserved for several months by keeping it in sealed, opaque containers at 5–10 °C and protected from light to prevent slow photo-oxidative degradation of PEDOT:PSS and the photoinitiator.

.


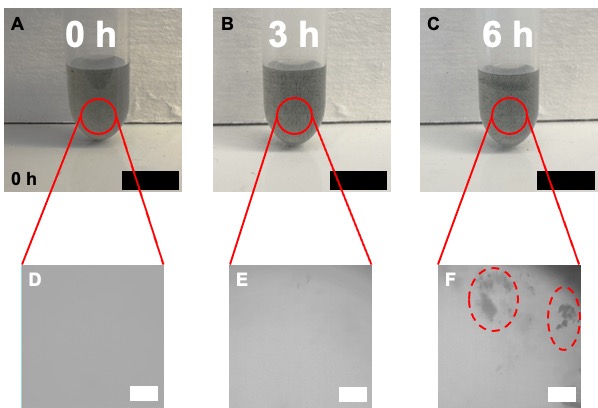


**Figure S4.** **Time-dependent stability and homogeneity of the composite conductive resin.** Optical micrographs of the composite resin (0.4 wt% OE, 0.15 wt% MWCNTs) taken at 0 h (A), 3 h (B), and 6 h (C) after preparation. Corresponding higher-magnification images are shown in panels D–F (scale bar: 1 mm). Red dotted circles highlight regions of emerging CNT aggregation, which become apparent at 6 h, indicating the onset of time-dependent resin instability.


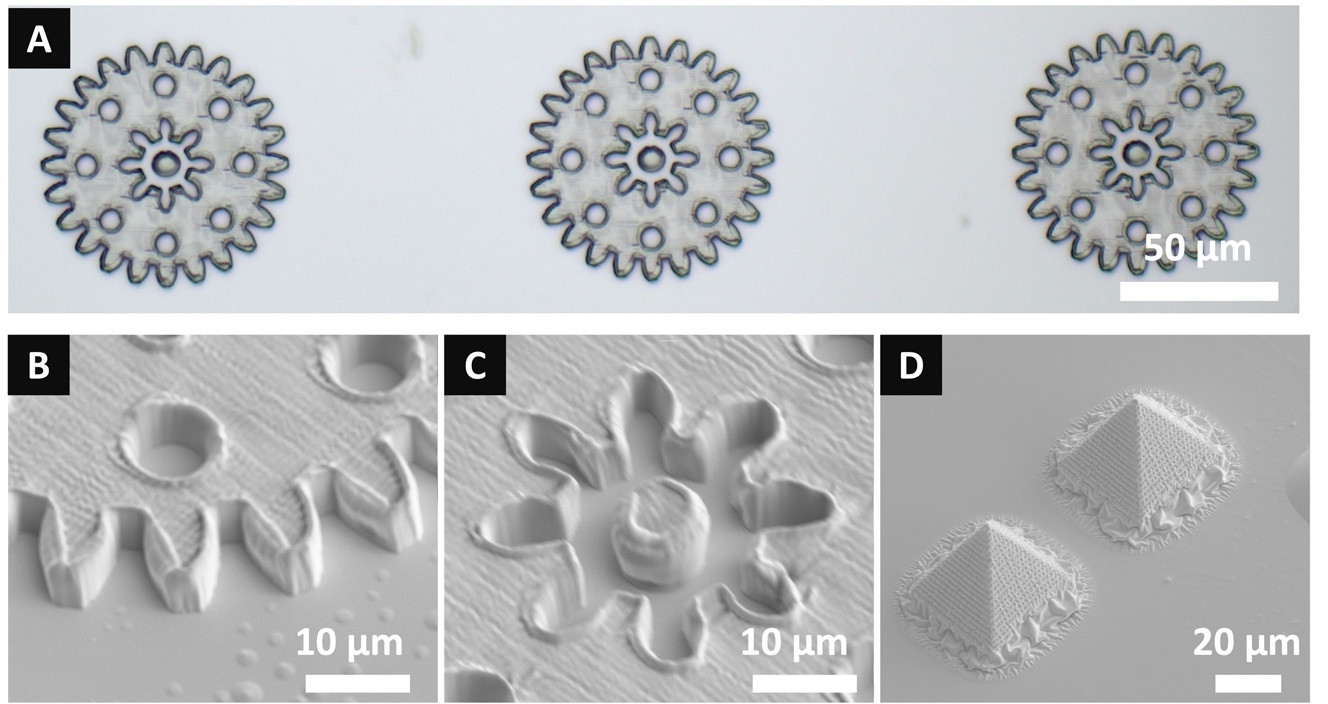


**Figure S5**. Optical micrograph of a micro-gear array.

Conductivity Measurement of Photosensitive Resins

**Figure S6.** **Electrical characterization of 2PP-fabricated microstructures with varying conductive filler content.** (A) Schematic of microbar fabrication (125 µm length, 20 µm width, 5 µm height) on a partially gold-coated glass substrate. Resins containing different filler compositions were used. (B–D) Representative I–V curves for microbars fabricated using resins with varying conductive filler content. Notably, the composite conductive resin containing 0.15 wt% MWCNTs and 0.4 wt% OE exhibited a significant increase in current response, indicating enhanced electrical conductivity at this formulation. Electrical conductivity was calculated using the equation.

$\sigma=\frac{G L}{A}$ (Equation S1)

where σ is electrical conductivity (S m^-1^), G is electrical conductance (S) and is derived from the slope of I-V curve, A is the cross-section area of the microstructure (20 µm × 5 µm) and l is the length of the microstructure (125 µm).


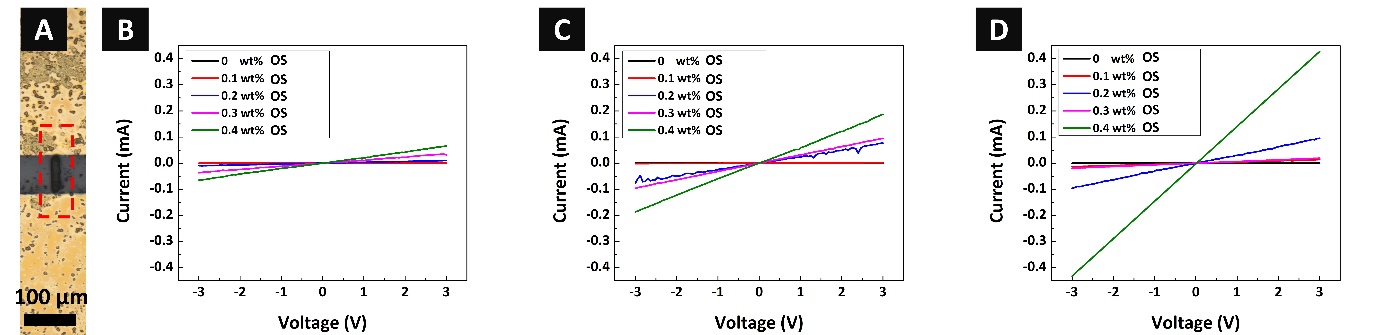

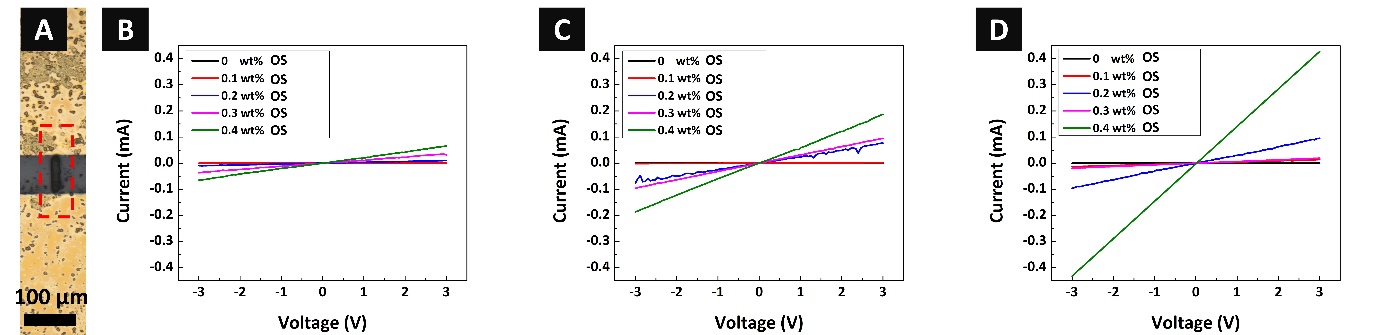


**OE**

**OE**

**OE**

**OE**

**OE**

**OE**

**OE**

**OE**

**OE**

**OE**

**OE**

**OE**

**OE**

**OE**

**OE**

**Current (A)**

**Current (A)**

**Current (A)**

**Figure S6.** **Electrical conductivity analysis of 2PP-fabricated microstructures using photosensitive resins with varying OE content.** (A) Optical micrograph of a 2PP-fabricated bar-shaped microstructure bridging two partially gold-coated regions on a glass coverslip. (B–D) Representative I–V curves acquired using a semiconductor device parameter analyzer. Each plot corresponds to a resin formulation with fixed MWCNT content and incremental OE concentrations: 0 wt% (black), 0.1 wt% (red), 0.2 wt% (blue), 0.3 wt% (magenta), and 0.4 wt% (green). (B) Resins containing 0.05 wt% MWCNTs, (C) 0.1 wt% MWCNTs, and (D) 0.15 wt% MWCNTs. Increasing OE content results in progressively higher current responses, demonstrating enhanced electrical performance of the composite resins at optimized dual-filler concentrations.

**
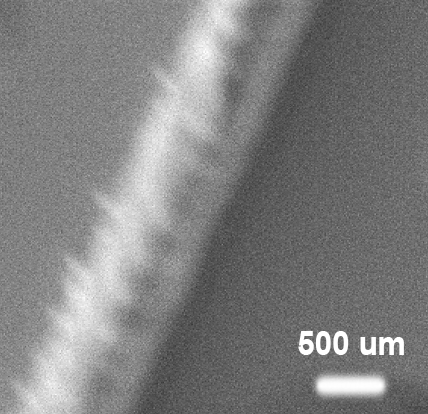
**

**Figure S7.** Scanning electron micrograph of a line patterned with the optimized composite resin, demonstrating a minimum achievable feature size of approximately ~700 nm.

**
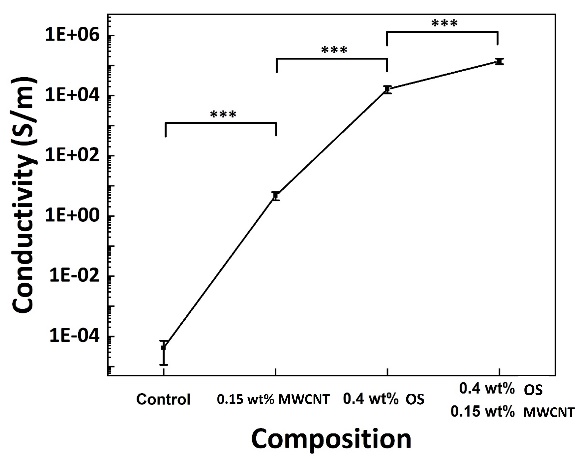
**

**0.4 wt% OE**

**0.15 wt% MWCNTs**

**0.4 wt% OE**

**Figure S8.** E**lectrical conductivity of 2PP-fabricated microbars based on four resin formulations:** (1) PEGDA-only control (no MWCNTs or OE), (2) 0.15 wt% MWCNTs, (3) 0.4 wt% OE, and (4) composite formulation containing 0.4 wt% OE and 0.15 wt% MWCNTs. The data demonstrate a clear synergistic enhancement in electrical conductivity when both conductive fillers are combined, outperforming the individual filler formulations as well as the polymer-only control.

**Table S5.** Electrical conductivity of 2PP-fabricated microstructures in the literature.

| Conductive filler | Preparation | Filler content (wt%) | Crosslinking polymer | Conductivity (S m^-1^) | Reference |
| --- | --- | --- | --- | --- | --- |
| SWCNT | Direct incorporation | 0.01 | Femtobond 4B | 0.07 | 40b |
| MWCNT | Direct incorporation | 0.2 | Acrylic | 46.8 | 20 |
| SWCNT | Direct incorporation | 5 |  | 0.00000097 | 40a |
| EDOT | Direct incorporation in resin, followed by in-situ chemical polymerization of EDOT into PEDOT after 2PP | 20 | PEGDA | 4 | 43 |
| CP | Direct incorporation in resin | 0.5 | PEGDA | 27000 | 21 |
| MWCNT + CP | Direct incorporation of MWCNT in resin, followed by in-situ self-assembly of CP after 2PP | 0.3 | Acryl Amide | 42.5 | 25a |
| MWCNT + CP | Direct incorporation of MWCNT in resin, followed by CP interpenetration after 2PP | 0.25 | PEGDA | 2.23 | 44 |
| HAuCl4 | Direct incorporation | 30 | SU-8 | 25000000 | 22 |
| HAuCl4 | Direct incorporation | 50 | PEG-triacry  (annealing) | 2200000 | 38a |
| HAuCl4 | Direct incorporation | 1.87 | SU-8 | 17241.38 | 38b |
| AgNO_3_ | Direct incorporation | 7.3 | Polyvinylpyrolidone | 2873563 | 39c |
| AgNWs | Direct incorporation | 0.4 | polyvinylcarbazole | 92.9 | 39a |
| AgBF_4_ | Direct incorporation | 0.2 | polyvinylcarbazole | 100000 | 39b |
| Graphene oxide | Direct incorporation | 0.1 | Photoresist | 0.0000985 | 23 |
| Graphene | Direct incorporation | 0.02 | Sol-gel | 0.00002891 | 41 |

Materials Confocal Microscopy (MCM)


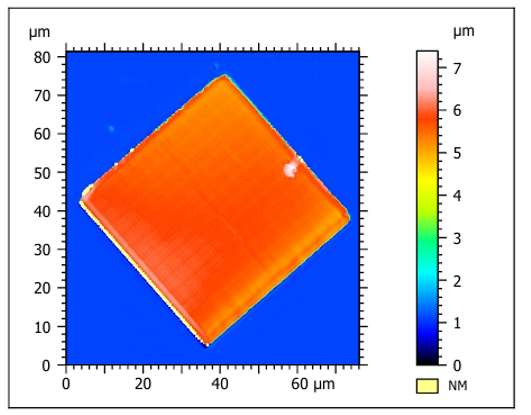


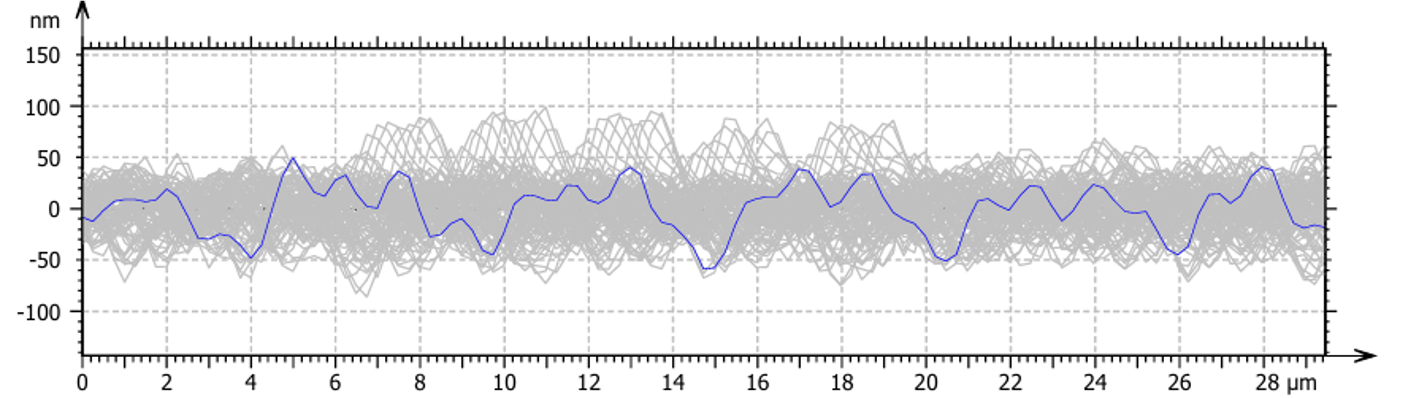


**Figure S9**. **Laser scanning confocal microscopy of a 2PP-fabricated polymer composite microcube.** Surface texture is visualized using a color-coded height map. A 50 µm × 50 µm region was extracted for quantitative roughness analysis, and the corresponding surface roughness profile is shown.

Calculation of Specific Capacitance

Specific capacitance (*C_SP_*) of the OEMWCNT microstructures was calculated using the following equation:

$C_{sp}= \frac{1}{2\Delta V\upsilon m}\int_{V_{1}}^{V_{2}} i dV$ (Equation S2)

, where $\upsilon$ is scan rate (0.1 V s^-1^), $A$ is surface area, $m$ is the mass of the microstructures, and $\Delta V$ is the potential sweep window. Mass of the microcapacitors was calculated based on the density of conductive-composite resin (*ρ*= 1.17 pg µm^-3^).

Calculation of Charge Storage Capacity:

The charge storage capacity (*Q*) of the OEMWCNT microstructures was calculated based on the following equation:

$Q=\frac{1}{\upsilon A}\int_{V_{1}}^{V_{2}} i dV$ (Equation S3)

, where $\upsilon$ is scan rate (0.1 V s^-1^), $A$ is surface area, and $\Delta V$ is the potential window ($\Delta V=1.2 V)$.

**
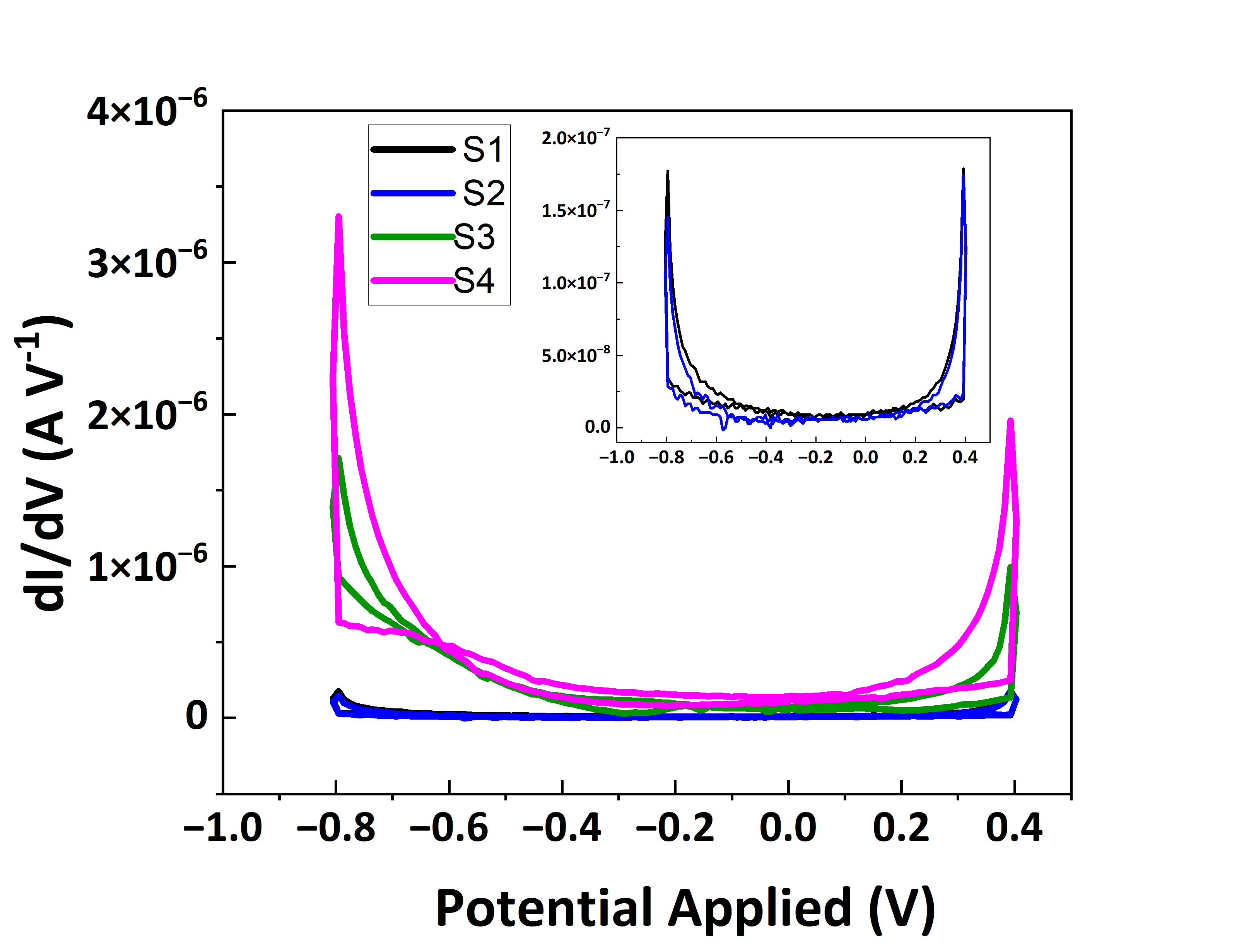
**

**Figure S10**. First derivative (dI/dV) of the CV shows a smooth, low baseline without narrow extrema, derivative processing

**Swelling and Degradation Studies:**

**
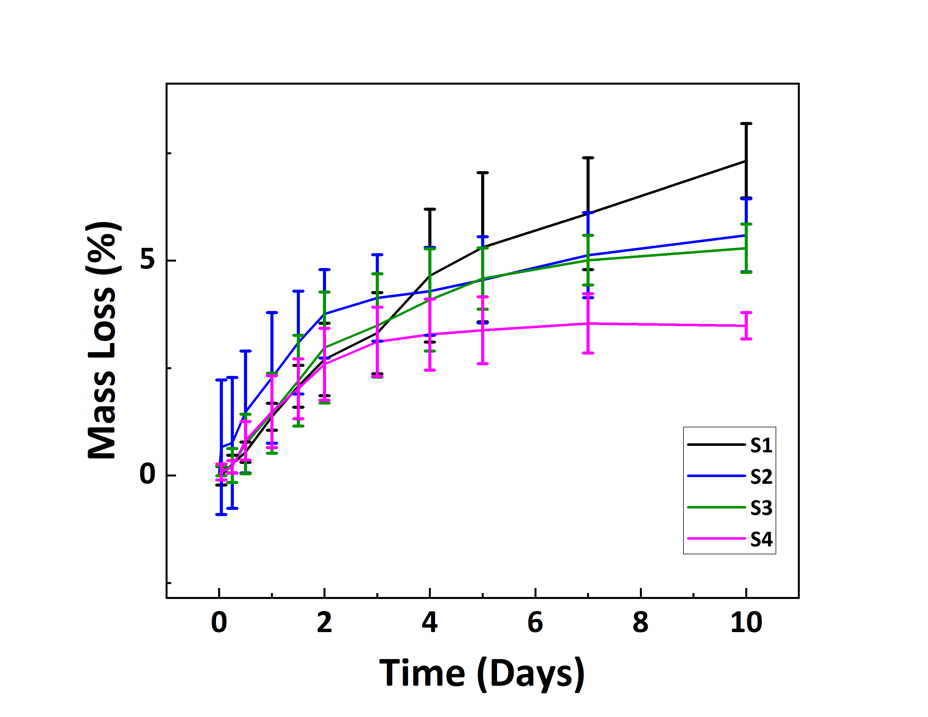
**

**Figure S11.** Mass loss as a function of time, S1 (PEGDA, black), S2 (PEGDA + 0.15 wt% MWCNTs, blue), S3 (PEGDA + 0.4 wt% OE, green), and S4 (PEGDA + 0.15 wt% MWCNTs + 0.4 wt% OE, pink) Data shown as mean ± S.E.M. (n=5). Statistical analysis revealed no significant differences in mass loss between groups (n = 5, p > 0.05), indicating that incorporation of conductive fillers did not adversely affect the hydrolytic stability of the PEGDA matrix

**Impedance stability over time**


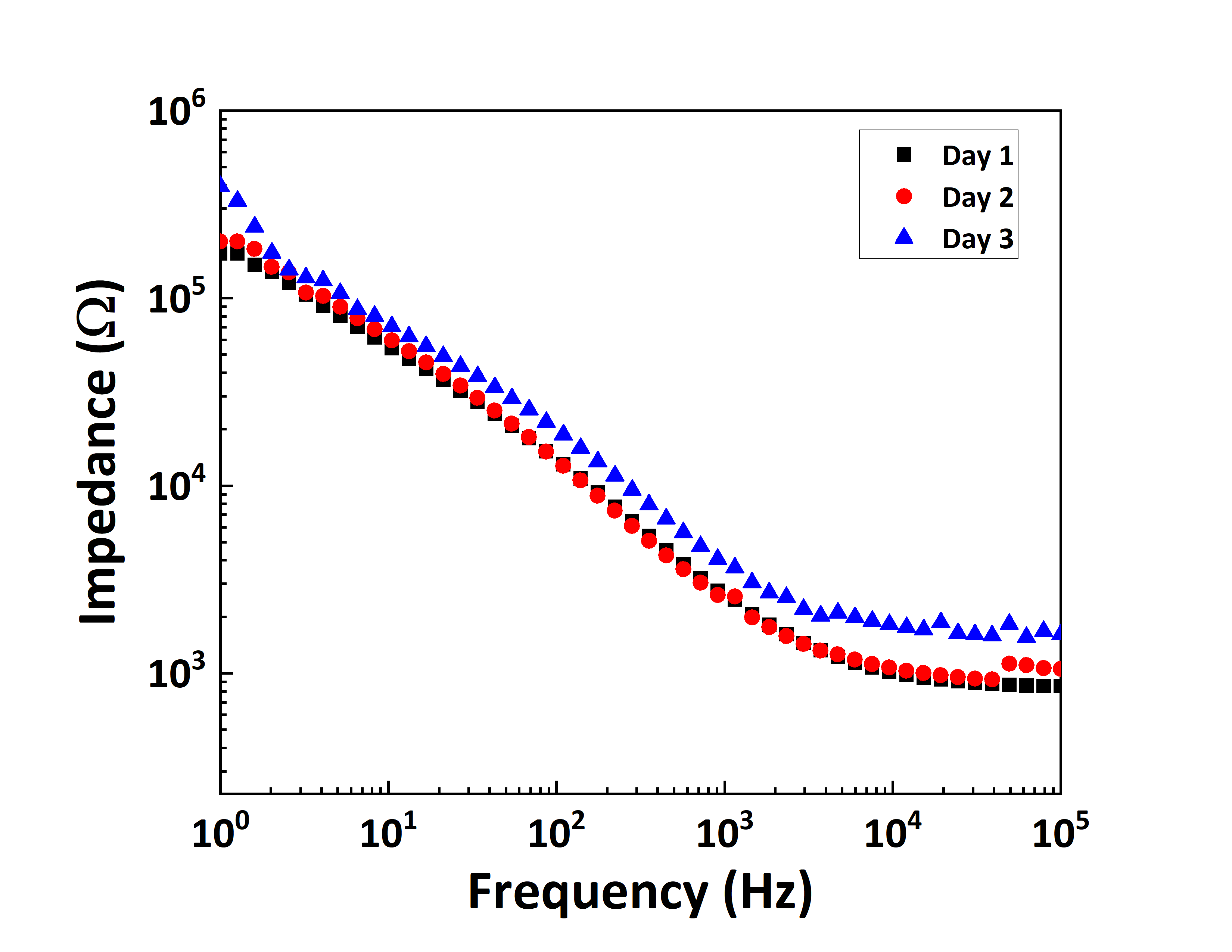


**Figure S12.** Impedance stability of the S4 (PEGDA + 0.15 wt% MWCNTs + 0.4 wt% OE, following incubation in PBS for 1, 2, and 3 days.


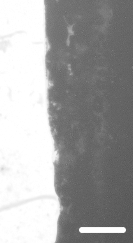

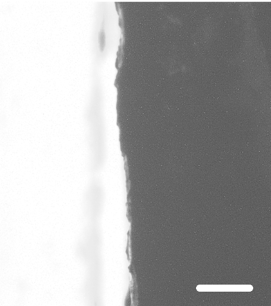

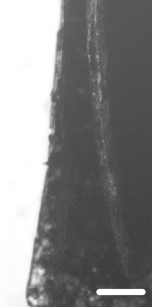

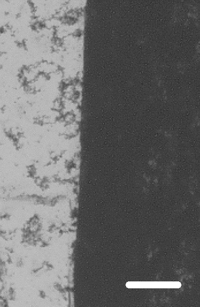

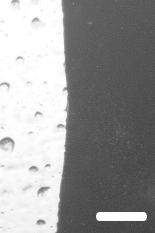

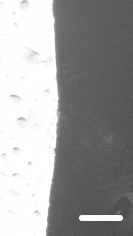

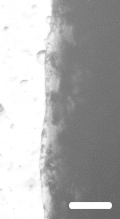

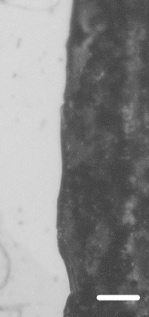


**A**

**B**

**C**

**D**

**E**

**F**

**G**

**H**

**Figure S13**. **Optical micrographs of OEMWCNT microstructures embedded in PDMS under stretching and bending deformation.** (A–C) Side-view images of samples during tensile testing: (A) before stretching, (B) at 50% strain, and (C) after 3000 stretching cycles at 10% strain. (D–H) Side-view images of samples during bending: (D) before deformation, (E) 500 µm deflection, (F) 1 mm deflection, (G) 2 mm deflection, and (H) 200 µm deflection after 500 bending cycles. Black regions correspond to OEMWCNT microstructures, and white regions correspond to the surrounding PDMS. Scale bar = 1 mm.
